# The impact of the COVID-19 pandemic on antimicrobial usage: an international patient-level cohort study

**DOI:** 10.1093/jacamr/dlaf037

**Published:** 2025-03-26

**Authors:** Refath Farzana, Stephan Jürgen Harbarth, Ly-Mee Yu, Edoardo Carretto, Catrin E Moore, Nicholas Alexander Feasey, Ana C Gales, Ushma Galal, Onder Ergonul, Dongeun Yong, Md Abdullah Yusuf, Balaji Veeraraghavan, Kenneth Chukwuemeka Iregbu, James Anton van Santen, Aghata Cardoso da Silva Ribeiro, Carolina Maria Fankhauser, Chisomo Judith Chilupsya, Christiane Dolecek, Diogo Boldim Ferreira, Fatihan Pinarlik, Jaehyeok Jang, Lal Sude Gücer, Laura Cavazzuti, Marufa Sultana, M D Nazmul Haque, Murielle Galas Haddad, Nubwa Medugu, Philip Ifeanyi Nwajiobi-Princewill, Roberta Marrollo, Rui Zhao, Vivekanandan B Baskaran, J V Peter, Sujith J Chandy, Yamuna Devi Bakthavatchalam, Timothy R Walsh, COVID-19/DRI Study Group, Katherine Paulina, Dhiviya Prabaa, Naveen Kumar Devanga Ragupathi, Alpay Azap, Ezgi Gülten, Elif Ateş, Özlem Azap, Nuran Sarı, İlkay Karaoğlan, Kübra Koçak, Özlem Mete, Emine Coşkun, Murat Kutlu, Şevval Özen Aksakal, Mehtap Aydın, Merve Çağlar Özer, Şirin Menekşe, Zeynep Ceren Karahan, Begüm Nalça Erdin, Cherkaoui Abdessalam, Nadia Colaizzi, Perince Fonton, Cyril Stucki, Riccardo Bianchi, Luca Bruni, Carlo Capatti, Souad Kassem, Francesco Navazio, Michela Paolucci, Benedetta Roatti, Khadija Abdulraheem, Tobechi Akujobi, Olanrewaju Falodun, Fortune Fibresima, Abid Anjum Abir, Sabah Arefin, Moumita Deb, Parsa Irin Disha, Farjana Akter Eva, K Rockybul Hasan, Anantu Islam, Halima Sadia Islam, Kimia Tuj Jannat, Zarin Sultana Liya, Fahmida Afroze Nadia, Afia Anjum Prova, Sadika Rahman, S M Rafiqul Islam, Arafat Sabbir, Soumik Talukder, Sultana Jahan Tuly, Lim Jones, Mandy Wootton

**Affiliations:** Department of Biology, Ineos Oxford Institute for Antimicrobial Research, University of Oxford, Oxford, UK; Faculty of Medicine, Geneva University Hospitals, Geneva, Switzerland; WHO Collaborating Center for Antimicrobial Resistance and Infection Control, Geneva, Switzerland; Nuffield Department of Primary Care Health Sciences, University of Oxford, Oxford, UK; Clinical Microbiology Laboratory, IRCCS Azienda Unità Sanitaria Locale, Reggio Emilia, Italy; Institute for Infection and Immunity, St George’s, University of London, London, UK; School of Medicine, University of St Andrews, St Andrews, Fife, UK; Malawi Liverpool Wellcome Programme, Kamuzu University of Health Sciences, Blantyre, Malawi; Division of Infectious Diseases, Universidade Federal de São Paulo, São Paulo, Brazil; Antimicrobial Resistance Institute of São Paulo (ARIES), São Paulo, Brazil; Nuffield Department of Primary Care Health Sciences, University of Oxford, Oxford, UK; Koc University İşbank Center for Infectious Diseases, Koc University, Istanbul, Türkiye; School of Medicine, Koç University, Istanbul, Türkiye; Department of Laboratory Medicine and Research Institute of Bacterial Resistance, Yonsei University College of Medicine, Seoul, South Korea; Department of Microbiology, National Institute of Neurosciences and Hospital, Dhaka, Bangladesh; Department of Clinical Microbiology, Christian Medical College, Vellore, India; Department of Microbiology, University of Abuja, Abuja, Nigeria; Department of Microbiology, National Hospital Abuja, Abuja, Nigeria; Nuffield Department of Primary Care Health Sciences, University of Oxford, Oxford, UK; Division of Infectious Diseases, Universidade Federal de São Paulo, São Paulo, Brazil; Antimicrobial Resistance Institute of São Paulo (ARIES), São Paulo, Brazil; Infection and Prevention Control, Geneva University Hospitals, Geneva, Switzerland; Malawi Liverpool Wellcome Programme, Kamuzu University of Health Sciences, Blantyre, Malawi; Centre for Tropical Medicine and Global Health, University of Oxford, Oxford, UK; Division of Infectious Diseases, Universidade Federal de São Paulo, São Paulo, Brazil; Koc University İşbank Center for Infectious Diseases, Koc University, Istanbul, Türkiye; Graduate School of Health Sciences, Koc University, Istanbul, Türkiye; Department of Laboratory Medicine and Research Institute of Bacterial Resistance, Yonsei University College of Medicine, Seoul, South Korea; Koc University İşbank Center for Infectious Diseases, Koc University, Istanbul, Türkiye; School of Medicine, Koç University, Istanbul, Türkiye; Hospital Hygiene Unit, IRCCS Azienda Unità Sanitaria Locale, Reggio Emilia, Italy; Department of Microbiology, National Institute of Neurosciences and Hospital, Dhaka, Bangladesh; Administration, Dhaka Medical College Hospital, Dhaka, Bangladesh; Administration, Dhaka Medical College Hospital, Dhaka, Bangladesh; Combined Military Hospital, Jashore Cantonment, Jashore, Bangladesh; Faculty of Medicine, Geneva University Hospitals, Geneva, Switzerland; Department of Microbiology, National Hospital Abuja, Abuja, Nigeria; Department of Microbiology, Nile University of Nigeria, Abuja, Nigeria; Department of Microbiology, National Hospital Abuja, Abuja, Nigeria; Clinical Microbiology Laboratory, IRCCS Azienda Unità Sanitaria Locale, Reggio Emilia, Italy; Nuffield Department of Primary Care Health Sciences, University of Oxford, Oxford, UK; Department of Clinical Microbiology, Christian Medical College, Vellore, India; Medical Intensive Care Unit, Christian Medical College, Vellore, India; Department of Pharmacology & Clinical Pharmacology, Christian Medical College, Vellore, India; Department of Clinical Microbiology, Christian Medical College, Vellore, India; Department of Biology, Ineos Oxford Institute for Antimicrobial Research, University of Oxford, Oxford, UK

## Abstract

**Background:**

This study aimed to evaluate the trends in antimicrobial prescription during the first 1.5 years of COVID-19 pandemic.

**Methods:**

This was an observational, retrospective cohort study using patient-level data from Bangladesh, Brazil, India, Italy, Malawi, Nigeria, South Korea, Switzerland and Turkey from patients with pneumonia and/or acute respiratory distress syndrome and/or sepsis, regardless of COVID-19 positivity, who were admitted to critical care units or COVID-19 specialized wards. The changes of antimicrobial prescription between pre-pandemic and pandemic were estimated using logistic or linear regression. Pandemic effects on month-wise antimicrobial usage were evaluated using interrupted time series analyses (ITSAs).

**Results:**

Antimicrobials for which prescriptions significantly increased during the pandemic were as follows: meropenem in Bangladesh (95% CI: 1.94–4.07) with increased prescribed daily dose (PDD) (95% CI: 1.17–1.58) and Turkey (95% CI: 1.09–1.58), moxifloxacin in Bangladesh (95% CI: 4.11–11.87) with increased days of therapy (DOT) (95% CI: 1.14–2.56), piperacillin/tazobactam in Italy (95% CI: 1.07–1.48) with increased DOT (95% CI: 1.01–1.25) and PDD (95% CI: 1.05–1.21) and azithromycin in Bangladesh (95% CI: 3.36–21.77) and Brazil (95% CI: 2.33–8.42). ITSA showed a significant drop in azithromycin usage in India (95% CI: −8.38 to −3.49 g/100 patients) and South Korea (95% CI: −2.83 to −1.89 g/100 patients) after WHO guidelines v1 release and increased meropenem usage (95% CI: 93.40–126.48 g/100 patients) and moxifloxacin (95% CI: 5.40–13.98 g/100 patients) in Bangladesh and sulfamethoxazole/trimethoprim in India (95% CI: 0.92–9.32 g/100 patients) following the Delta variant emergence.

**Conclusions:**

This study reinforces the importance of developing antimicrobial stewardship in the clinical settings during inter-pandemic periods.

## Introduction

At the beginning of the COVID-19 pandemic, there was a sudden and significant change in clinical practice and health-seeking behaviour globally,^1,2^ accompanied by the lack of and/or misinterpretations of scientific evidence for COVID-19 treatment and preventive measures.^2^ Healthcare systems quickly became overloaded and increasingly fragile due to the vast numbers of patients requiring critical care concurrently.^3^ Throughout the COVID-19 pandemic, healthcare workers also contracted the disease, sometimes becoming too unwell to work, and even when mildly unwell, subject to enforced isolation leading to a significant reduction in staff-to-patient ratios. Limited access to diagnostic tests and personal protective equipment with improvizations to infection prevention and control (IPC) practices led to higher nosocomial transmission of MDR organisms during the pandemic.^2–5^ Justification for empiric broad-spectrum antimicrobial usage was made, given that COVID-19 infection leads to the dysregulation of the immune system, whereby the patients may be vulnerable to secondary bacterial and fungal infections.^6^ Studies also reported indiscriminate antimicrobial consumption without microbiological evidence of bacterial infections, particularly during the early phase of the pandemic when data on secondary infections were sparse and often contradictory.^7,8^

As of December 2021, the excess mortality due to the COVID-19 pandemic globally was estimated to be 14.9 million, but the death rate varied markedly between countries.^9,10^ Antimicrobial use fluctuated in the different pandemic waves, depending on multiple factors in different countries with heterogeneity in the choice of antimicrobials.^11–13^ Despite many single-centre or single-country studies (mostly using aggregate-level data), few studies compared antibiotic usage patterns and trends between multiple countries using individual patient-level data. Variation between countries in antibiotic treatment protocols and decision-making during the pandemic is yet to be determined globally.^11,14^

We performed an international cohort study to assess the impact of the COVID-19 pandemic on antibiotic-prescribing practices in clinical settings with diverse patient management policies using individual patient-level data through a global network involving 17 hospitals from 9 countries spanning high, middle- and low-income countries.^15^

## Methods

### Study design

This observational, retrospective cohort study included tertiary care institutions in Bangladesh, Brazil, India, Italy, Malawi, Nigeria, South Korea, Switzerland and Turkey. The countries were selected based on the following criteria: (i) a balance of low (Malawi), lower-middle (Bangladesh, India and Nigeria), upper-middle (Brazil and Turkey) and high (Italy, South Korea and Switzerland) income countries^15^; (ii) variations in dates in the first case of COVID-19 (Figure S1, available as Supplementary data at *JAC-AMR* Online)^10^ and (iii) varied levels of reported cases of COVID-19 and associated deaths (Figure S2).^10^ We approached one site per country, but on engagement, we were able to enrol additional sites from Bangladesh (*n* = 3) and Turkey (*n* = 7) with an aim of including 100 patients per month per country.

The consortium for this project included a mixture of established collaborators and new partners to optimize geographical reach. The sites were chosen based on discussions with the collaborators about whether their hospital infrastructure during the pandemic could support data collection for this study. All participating sites were national referral hospitals for suspected COVID-19 patients (Table S1). The hospitals demonstrated varying levels of clinical microbiological capacity and antimicrobial stewardship (AMS) activities (Table S2), as well as distinct infection management strategies based on policy, infrastructure and facilities (Table S3).

### Case ascertainment

**Figure 1.**
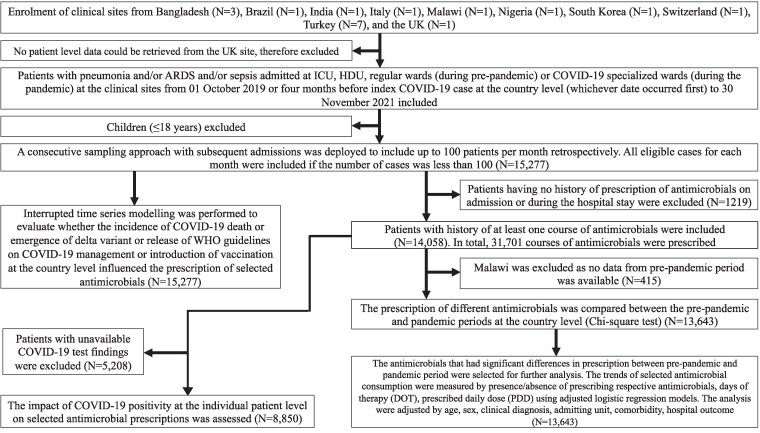
Study flow diagram.

Where available, we abstracted data from 01 October 2019, or 4 months before the index COVID-19 case at the country level (whichever date occurred first), up to 30 November 2021. The index confirmed cases of COVID-19 occurred between 20 January 2020 and 02 April 2020 in all enrolled sites (Figure 1). Patients were eligible for this study if admitted to intensive care, intermediate care or specialized COVID isolation wards, presenting with pneumonia and/or acute respiratory distress syndrome (ARDS) and/or sepsis related to an infectious syndrome beyond the respiratory tract (e.g. meningitis, urosepsis, peritonitis, endocarditis, cellulitis, etc.) irrespective of COVID-19 positivity, some transited through the emergency department. ICUs included specialist units involving treatment and monitoring with ventilators, monitoring equipment, intravenous lines, pumps, feeding tubes, drains and catheters for seriously ill patients. High-dependency units (HDUs) were the step-down units between ICUs and general wards providing intermediate care. Specialized COVID isolation wards were wards that provided care to COVID-19 patients only. Children (≤18 years) were excluded. Patients fell into the pre-pandemic period if they were admitted to the hospital before the report of a COVID-19 index case at the country level and into the pandemic group if they were admitted on or after the date of the index case. A patient’s COVID-19 status, whether positive or negative, was determined based on the results of COVID-19 testing only. Further details on eligibility criteria of the study participants can be found in Text S1.

A consecutive sampling approach with subsequent admissions was deployed to include up to 100 patients per month. All eligible cases for each month were included if the number of cases was <100. To ensure representation from multiple sites in Bangladesh and Turkey, 25 patients were recruited per site in Bangladesh and 10 per site in Turkey, targeting a total of up to 100 patients monthly in each country. This project was approved by the appropriate ethical bodies at the collaborating sites (Table S4).

Clinical sites were involved with patient selection and data collection retrospectively. Retrospective data collection meant patients’ selection was performed based on the clinical findings in the hospital records. Patients’ data included admission details (date of admission, admitting hospital unit, date of discharge or death and hospital outcome), demographic data (age and sex) and clinical information (clinical presentation at the onset of pneumonia, ARDS or sepsis, antibiotic treatment including duration and dose, COVID-19 positivity and comorbidities such as chronic cardiac disease, hypertension, chronic pulmonary disease including asthma, diabetes, active tuberculosis and obesity). Additionally, we collected data on hydroxychloroquine prescription from the eligible patients. Data were collected and managed through the Research Electronic Data Capture tools hosted at the University of Oxford.^16^ Although one hospital per country was enrolled mostly for this study, the ‘name of the country’ is used throughout the paper for data representation only.

### Statistical analysis

The primary objective was to evaluate the changes in antimicrobial prescriptions between pre-pandemic and pandemic periods. An initial chi-squared test was performed to explore the association between the COVID-19 pandemic and individual antibiotic prescribing at enrolled sites. We explored differences in antimicrobial prescription before and during the pandemic and differences in antimicrobial prescriptions between individuals that were COVID-19 positive and COVID-19 negative. Binary logistic regression was used to model the data for both the sets of comparisons.

For all antimicrobials with prescriptions, changes in days of therapy (DOT) and prescribed daily dose (PDD) were compared using linear regression models. DOT for each individual antimicrobial course was calculated by determining the duration between the initiation and cessation dates of administration. PDD was derived by multiplying the number of doses prescribed per day and the strength per dose. The models were run on log-transformed outcomes, as the data were skewed, with the results back-transformed for interpretation as geometric mean ratios (GMRs).

All logistic and linear regression models were run separately for each country and adjusted for age (continuous), sex (male/female), admitting wards [ICU/HDU/Department of Critical Care (DCC)/COVID specialized, including regular wards for the pre-pandemic period], comorbidities (binary for present/absent), patients’ hospital outcome (died/discharged alive) and diagnosis type (sepsis only/pneumonia only/ARDS only/sepsis and pneumonia/sepsis and ARDS/pneumonia and ARDS/sepsis, pneumonia and ARDS).

Interrupted time series analyses (ITSAs) were performed to assess the effect of COVID-19-associated deaths, detect the Delta variant and the release of WHO guidelines on antimicrobial prescribing for antimicrobials that had significant differences in usage between pre-pandemic and pandemic at country level during the pandemic period, i.e. from the month of the introduction of index case at the country level to 30 November 2021. Antimicrobial prescribing was defined by the total PDD (in grams) of respective antimicrobials (continuous outcome), per 100 patients, for each month, and was analysed using Prais–Winsten AR(1) regression. The models were fitted with robust (to heteroscedasticity) estimators for the variance–covariance matrix, which uses the Huber/White/sandwich estimator. No predictors were included in these models, aside from the factor representing the ‘interruption’.

Further chi-squared (or Fisher’s exact) tests were performed to assess the associations between other binary factors described in the paper. As these analyses were exploratory, adjustment for multiple testing was not carried out.

Statistical significance was set at *P *< 0.05. Statistical analyses were performed using STATA 18.0 Standard Edition (StataCorp, College Station, TX, USA) and IBM SPSS Statistics (v29.0.0.0). Graphs were generated using Tableau Desktop (v2024.2).

## Results

### Baseline characteristics of the study population

An estimated 14 058 patients were prescribed 31 701 courses of antimicrobials, of which 10  579 (33.4%) prescriptions were made on admission and 20 656 (65.2%) during their hospital stay, with 466 (1.5%) missing data on start and/or stop dates of administration (Table 1). The mean number of antimicrobials prescribed per patient in each country ranged from 1 to 3. The mean duration between the patient’s hospital admission and the first antimicrobial prescription ranged from 0.2 to 11.1 days (Table 1).

**Table 1.** Characteristics of the overall study population

		Bangladesh		Brazil		India		Italy		Malawi^a^	Nigeria		South Korea		Switzerland		Turkey	
		Pre-pandemic (*N* = 416)	Pandemic (*N* = 1780)	Pre-pandemic (*N* = 161)	Pandemic (*N* = 1131)	Pre-pandemic (*N* = 760)	Pandemic (*N* = 1837)	Pre-pandemic (*N* = 490)	Pandemic (*N* = 1902)	Pandemic (*N* = 415)	Pre-pandemic (*N* = 17)	Pandemic (*N* = 468)	Pre-pandemic (*N* = 246)	Pandemic (*N* = 1789)	Pre-pandemic (*N* = 259)	Pandemic (*N* = 932)	Pre-pandemic (*N* = 279)	Pandemic (*N* = 1176)
Age (years)	Mean (SD)	55.8 (15.9)	51.7 (16.0)	58.7 (18.8)	60.6 (15.6)	46.8 (16.4)	47.2 (15.4)	74.5 (15.4)	74.4 (14.8)	50.8 (16.4)	45.4 (16.8)	51.6 (15.8)	65.8 (14.2)	66.5 (14.9)	69.8 (14.6)	68.0 (17.3)	64.2 (16.2)	66.0 (15.5)
	18–30, *n* (%)	40 (9.6)	233 (13.1)	17 (10.6)	50 (4.4)	172 (22.6)	337 (18.3)	6 (1.2)	19 (1.0)	51 (12.3)	5 (29.4)	57 (12.2)	6 (2.4)	59 (3.3)	6 (2.3)	24 (2.6)	14 (5.0)	42 (3.6)
	31–40, *n* (%)	38 (9.1)	281 (15.8)	12 (7.5)	82 (7.3)	84 (11.1)	283 (15.4)	14 (2.9)	39 (2.1)	77 (18.6)	0 (0.0)	61 (13.0)	10 (4.1)	62 (3.5)	4 (1.5)	53 (5.7)	8 (2.9)	40 (3.4)
	41–50, *n* (%)	57 (13.7)	250 (14.0)	23 (14.3)	128 (11.3)	154 (20.3)	384 (20.9)	19 (3.9)	96 (5.0)	80 (19.3)	3 (17.6)	81 (17.3)	16 (6.5)	111 (6.2)	14 (5.4)	72 (7.7)	28 (10.0)	76 (6.5)
	>50, *n* (%)	279 (67.1)	1013 (56.9)	109 (67.7)	871 (77.0)	344 (45.3)	816 (44.4)	451 (92.0)	1747 (91.9)	207 (49.9)	8 (47.1)	263 (56.2)	214 (87.0)	1537 (85.9)	235 (90.7)	783 (84.0)	229 (82.1)	1018 (86.6)
	*Missing, n (%)*	2 (0.5)	3 (0.2)	0 (0.0)	0 (0.0)	6 (0.8)	17 (0.9)	0 (0.0)	1 (0.1)	0 (0.0)	1 (5.9)	6 (1.3)	0 (0.0)	20 (1.1)	0 (0.0)	0 (0.0)	0 (0.0)	0 (0.0)
Sex	Male, *n* (%)	410 (98.6)	1212 (68.1)	86 (53.4)	679 (60.0)	481 (63.3)	1133 (61.7)	286 (58.4)	1099 (57.8)	269 (64.8)	13 (76.5)	319 (68.2)	159 (64.6)	1123 (62.8)	154 (59.5)	556 (59.7)	158 (56.6)	693 (58.9)
	Female, *n* (%)	6 (1.4)	568 (31.9)	75 (46.6)	442 (39.1)	278 (36.6)	701 (38.2)	204 (41.6)	801 (42.1)	146 (35.2)	4 (23.5)	149 (31.8)	87 (35.4)	664 (37.1)	105 (40.5)	375 (40.2)	118 (42.3)	478 (40.6)
	Other, *n* (%)	0 (0.0)	0 (0.0)	0 (0.0)	10 (0.9)	1 (0.1)	3 (0.2)	0 (0.0)	2 (0.1)	0 (0.0)	0 (0.0)	0 (0.0)	0 (0.0)	0 (0.0)	0 (0.0)	1 (0.1)	2 (0.7)	5 (0.4)
	*Missing, n (%)*	0 (0.0)	0 (0.0)	0 (0.0)	0 (0.0)	0 (0.0)	0 (0.0)	0 (0.0)	0 (0.0)	0 (0.0)	0 (0.0)	0 (0.0)	0 (0.0)	2 (0.1)	0 (0.0)	0 (0.0)	1 (0.4)	0 (0.0)
Clinical diagnosis	Sepsis only, *n* (%)	77 (18.5)	9 (0.5)	67 (41.6)	159 (14.1)	346 (45.5)	753 (41.0)	364 (74.3)	1105 (58.1)	0 (0.0)	3 (17.6)	17 (3.6)	45 (18.3)	400 (22.4)	116 (44.8)	116 (12.4)	179 (64.2)	799 (67.9)
	Pneumonia only, *n* (%)	303 (72.8)	1652 (92.8)	29 (18.0)	180 (15.9)	213 (28.0)	268 (14.6)	65 (13.3)	463 (24.3)	212 (51.1)	14 (82.4)	408 (87.2)	152 (61.8)	1014 (56.7)	89 (34.4)	604 (64.8)	19 (6.8)	61 (5.2)
	ARDS only, *n* (%)	1 (0.2)	75 (4.2)	0 (0.0)	29 (2.6)	58 (7.6)	288 (15.7)	0 (0.0)	23 (1.2)	37 (8.9)	0 (0.0)	21 (4.5)	1 (0.4)	17 (1.0)	31 (12.0)	37 (4.0)	0 (0.0)	8 (0.7)
	Sepsis and pneumonia, *n* (%)	33 (7.9)	37 (2.1)	59 (36.6)	94 (8.3)	49 (6.4)	62 (3.4)	27 (5.5)	101 (5.3)	0 (0.0)	0 (0.0)	10 (2.1)	43 (17.5)	284 (15.9)	21 (8.1)	35 (3.8)	81 (29.0)	263 (22.4)
	Sepsis and ARDS, *n* (%)	0 (0.0)	1 (0.1)	4 (2.5)	176 (15.6)	14 (1.8)	39 (2.1)	16 (3.3)	27 (1.4)	0 (0.0)	0 (0.0)	0 (0.0)	2 (0.8)	10 (0.6)	2 (0.8)	2 (0.2)	0 (0.0)	3 (0.3)
	Pneumonia and ARDS, *n* (%)	1 (0.2)	6 (0.3)	0 (0.0)	305 (27.0)	70 (9.2)	396 (21.6)	14 (2.9)	159 (8.4)	166 (40.0)	0 (0.0)	11 (2.4)	2 (0.8)	48 (2.7)	0 (0.0)	123 (13.2)	0 (0.0)	5 (0.4)
	Sepsis, pneumonia and ARDS, *n* (%)	1 (0.2)	0 (0.0)	2 (1.2)	188 (16.6)	10 (1.3)	31 (1.7)	4 (0.8)	24 (1.3)	0 (0.0)	0 (0.0)	1 (0.2)	1 (0.4)	16 (0.9)	0 (0.0)	15 (1.6)	0 (0.0)	37 (3.1)
Admitting ward^b^	ICU, *n* (%)	5 (1.2)	314 (17.6)	104 (64.6)	490 (43.3)	393 (51.7)	1154 (62.8)	5 (1.0)	23 (1.2)	0 (0.0)	15 (88.2)	2 (0.4)	246 (100.0)^e^	1669 (93.3)^e^	152 (58.7)	195 (20.9)	262 (93.9)	1051 (89.4)
	HDU, *n* (%)	3 (0.7)	25 (1.4)	57 (35.4)	64 (5.7)	364 (47.9)	676 (36.8)	33 (6.7)	106 (5.6)	415 (100.0)	2 (11.8)	0 (0.0)	0 (0.0)	0 (0.0)	107 (41.3)	206 (22.1)	0 (0.0)	5 (0.4)
	DCC, *n* (%)	1 (0.2)	0 (0.0)	0 (0.0)	1 (0.1)	3 (0.4)	6 (0.3)	452 (92.2)	1362 (71.6)	0 (0.0)	0 (0.0)	0 (0.0)	0 (0.0)	0 (0.0)	0 (0.0)	5 (0.5)	16 (5.7)	34 (2.9)
	Regular/COVID specialized, *n* (%)	407 (97.8)	1441 (81.0)	0 (0.0)	576 (50.9)	0 (0.0)	1 (0.1)	0 (0.0)	411 (21.6)	0 (0.0)	0 (0.0)	466 (99.6)	0 (0.0)	120 (6.7)	0 (0.0)	526 (56.4)	1 (0.4)	86 (7.3)
	*Missing, n (%)*	0 (0.0)	0 (0.0)	0 (0.0)	0 (0.0)	0 (0.0)	0 (0.0)	0 (0.0)	0 (0.0)	0 (0.0)	0 (0.0)	0 (0.0)	0 (0.0)	0 (0.0)	0 (0.0)	0 (0.0)	0 (0.0)	0 (0.0)
COVID-19 result	Positive, *n* (%)	0 (0.0)	470 (26.4)	0 (0.0)	1025 (90.6)	0 (0.0)	430 (23.4)	0 (0.0)	432 (22.7)	321 (77.3)	0 (0.0)	358 (76.5)	0 (0.0)	63 (3.5)	0 (0.0)	767 (82.3)	0 (0.0)	246 (20.9)
	Negative, *n* (%)	0 (0.0)	214 (12.0)	0 (0.0)	77 (6.8)	3 (0.4)	566 (30.8)	0 (0.0)	1301 (68.4)	84 (20.2)	6 (35.3)	35 (7.5)	9 (3.7)	1588 (88.8)	0 (0.0)	85 (9.1)	33 (11.8)	710 (60.4)
	Undetermined, *n* (%)	0 (0.0)	2 (0.1)	0 (0.0)	0 (0.0)	0 (0.0)	1 (0.1)	0 (0.0)	3 (0.2)	2 (0.5)	0 (0.0)	15 (3.2)	0 (0.0)	0 (0.0)	0 (0.0)	3 (0.3)	0 (0.0)	1 (0.1)
	*Missing* ^c^, *n (%)*	416 (100.0)	1094 (61.5)	161 (100.0)	29 (2.6)	757 (99.6)	840 (45.7)	490 (100.0)	166 (8.7)	8 (1.9)	11 (64.7)	60 (12.8)	237 (96.3)	138 (7.7)	259 (100.0)	77 (8.3)	246 (88.2)	219 (18.6)
Comorbidities	Chronic cardiac disease (excl. hypertension), *n* (%)	76 (18.3)	142 (8.0)	34 (21.1)	272 (24.0)	14 (1.8)	46 (2.5)	177 (36.1)	614 (32.3)	70 (16.9)	3 (17.6)	8 (1.7)	55 (22.4)	414 (23.1)	83 (32.0)	259 (27.8)	135 (48.4)	486 (41.3)
	Hypertension, *n* (%)	117 (28.1)	528 (29.7)	67 (41.6)	728 (64.4)	198 (26.1)	488 (26.6)	259 (52.9)	946 (49.7)	69 (16.6)	4 (23.5)	150 (32.1)	87 (35.4)	687 (38.4)	113 (43.6)	473 (50.8)	127 (45.5)	562 (47.8)
	Chronic obstructive pulmonary disease, *n* (%)	128 (30.8)	69 (3.9)	21 (13.0)	138 (12.2)	19 (2.5)	8 (0.4)	81 (16.5)	228 (12.0)	8 (1.9)	2 (11.8)	4 (0.9)	24 (9.8)	456 (25.5)	59 (22.8)	122 (13.1)	53 (19.0)	126 (10.7)
	Asthma, *n* (%)	23 (5.5)	138 (7.8)	5 (3.1)	37 (3.3)	13 (1.7)	36 (2.0)	6 (1.2)	16 (0.8)	27 (6.5)	0 (0.0)	13 (2.8)	9 (3.7)	106 (5.9)	17 (6.6)	65 (7.0)	14 (5.0)	47 (4.0)
	Diabetes, *n* (%)	101 (24.3)	527 (29.6)	47 (29.2)	406 (35.9)	212 (27.9)	611 (33.3)	128 (26.1)	464 (24.4)	77 (18.6)	0 (0.0)	99 (21.2)	59 (24.0)	491 (27.4)	65 (25.1)	231 (24.8)	92 (33.0)	343 (29.2)
	Tuberculosis (active), *n* (%)	13 (3.1)	10 (0.6)	6 (3.7)	10 (0.9)	23 (3.0)	40 (2.2)	0 (0.0)	1 (0.1)	18 (4.3)	2 (11.8)	5 (1.1)	12 (4.9)	31 (1.7)	1 (0.4)	3 (0.3)	0 (0.0)	1 (0.1)
	Obesity, *n* (%)	1 (0.2)	3 (0.2)	10 (6.2)	190 (16.8)	1 (0.1)	8 (0.4)	51 (10.4)	201 (10.6)	0 (0.0)	0 (0.0)	5 (1.1)	1 (0.4)	4 (0.2)	24 (9.3)	193 (20.7)	15 (5.4)	52 (4.4)
	Patients without any comorbidity stated above, *n* (%)	325 (78.1)	440 (24.7)	103 (64.0)	548 (48.5)	125 (16.4)	192 (10.5)	277 (56.5)	903 (47.5)	234 (56.4)	6 (35.3)	49 (10.5)	2 (0.8)	0 (0.0)	175 (67.6)	575 (61.7)	147 (52.7)	747 (63.5)
Antimicrobials prescribed	Mean number prescribed (SD)	2.0 (1.1)	2.0 (1.4)	2.4 (1.5)	3.2 (1.9)	1.6 (0.7)	1.7 (0.6)	2.2 (1.8)	1.8 (1.3)	1.0 (0.0)	2.5 (0.9)	2.0 (0.6)	2.9 (1.6)	3.1 (1.8)	2.0 (1.0)	2.2 (1.5)	3.0 (1.8)	2.9 (1.8)
	At least one antimicrobials prescribed, *n* (%)	416 (100.0)	1780 (100.0)	161 (100.0)	1131 (100.0)	760 (100.0)	1837 (100.0)	490 (100.0)	1902 (100.0)	415 (100.0)	17 (100.0)	468 (100.0)	246 (100.0)	1789 (100.0)	259 (100.0)	932 (100.0)	279 (100.0)	1176 (100.0)
	Two antimicrobials simultaneously, *n* (%)	174 (66.7)	561 (63.0)	74 (59.2)	413 (44.5)	274 (87.8)	719 (92.8)	133 (62.1)	514 (72.3)	0 (.)	11 (68.8)	344 (85.8)	63 (38.0)	432 (33.6)	100 (66.2)	270 (60.8)	104 (45.8)	354 (40.8)
	Three antimicrobials simultaneously, *n* (%)	57 (21.8)	167 (18.7)	33 (26.4)	158 (17.0)	28 (9.0)	46 (5.9)	40 (18.7)	115 (16.2)	0 (.)	2 (12.5)	48 (12.0)	39 (23.5)	290 (22.6)	39 (25.8)	73 (16.4)	52 (22.9)	218 (25.1)
	More than three antimicrobials simultaneously, *n* (%)	30 (11.5)	163 (18.3)	18 (14.4)	358 (38.5)	10 (3.2)	10 (1.3)	41 (19.2)	82 (11.5)	0 (.)	3 (18.8)	9 (2.2)	64 (38.6)	563 (43.8)	12 (7.9)	101 (22.7)	71 (31.3)	295 (34.0)
	*Missing* ^d^, *n (%)*	35 (8.4)	345 (19.4)	0 (0.0)	52 (4.6)	0 (0.0)	0 (0.0)	0 (0.0)	0 (0.0)	415 (100.0)	0 (0.0)	5 (1.1)	0 (0.0)	1 (0.1)	0 (0.0)	8 (0.9)	0 (0.0)	0 (0.0)
Duration between admission and first antimicrobial administration (days)	Mean (SD)	0.2 (1.0)	0.4 (2.4)	4.4 (13.7)	1.9 (4.8)	3.4 (11.7)	2.5 (4.9)	4.2 (36.7)	3.4 (19.8)	. (.)	0.4 (0.7)	1.9 (24.1)	6.4 (13.4)	6.5 (14.6)	3.2 (6.6)	3.0 (8.5)	11.1 (32.7)	6.6 (12.9)
Hospital outcome	Died, *n* (%)	77 (18.5)	232 (13.0)	66 (41.0)	459 (40.6)	236 (31.1)	683 (37.2)	59 (12.0)	324 (17.0)	85 (20.5)	1 (5.9)	48 (10.3)	106 (43.1)	744 (41.6)	46 (17.8)	183 (19.6)	174 (62.4)	732 (62.2)
	Discharged alive, *n* (%)	223 (53.6)	1115 (62.6)	94 (58.4)	659 (58.3)	484 (63.7)	1049 (57.1)	425 (86.7)	1572 (82.6)	329 (79.3)	14 (82.4)	406 (86.8)	140 (56.9)	1035 (57.9)	212 (81.9)	746 (80.0)	93 (33.3)	372 (31.6)
	Discharged against medical advice, *n* (%)	81 (19.5)	419 (23.5)	0 (0.0)	12 (1.1)	40 (5.3)	104 (5.7)	6 (1.2)	5 (0.3)	1 (0.2)	1 (5.9)	13 (2.8)	0 (0.0)	2 (0.1)	1 (0.4)	3 (0.3)	5 (1.8)	57 (4.8)
	*Missing, n (%)*	35 (8.4)	14 (0.8)	1 (0.6)	1 (0.1)	0 (0.0)	1 (0.1)	0 (0.0)	1 (0.1)	0 (0.0)	1 (5.9)	1 (0.2)	0 (0.0)	8 (0.4)	0 (0.0)	0 (0.0)	7 (2.5)	15 (1.3)
Duration of hospital stay (days)	Mean (SD)	5.0 (6.4)	9.5 (11.9)	23.4 (24.7)	20.5 (21.3)	21.0 (18.8)	20.6 (21.2)	14.2 (10.8)	15.9 (11.8)	5.9 (6.7)	11.6 (11.2)	11.2 (30.5)	51.5 (61.1)^e^	46.0 (52.8)^e^	24.6 (34.2)	21.0 (22.3)	37.1 (42.4)	26.4 (30.2)

^a^Pre-pandemic data were not available. Details of individual-level antimicrobial usage and consumption were not routinely captured at Queen Elizabeth Central Hospital before the implementation of the International Severe Acute Respiratory and Emerging Infection Consortium (ISARIC) protocol during the COVID-19 pandemic.

^b^There are observations of 408 patients admitted to wards rather than in ICU or HDU in the pre-pandemic period (407 patients from Bangladesh and 1 from Turkey). These are considered as regular wards.

^c^Test during the pre-pandemic period was not performed for the patients.

^d^Missing data on the start and stop date of antimicrobials.

^e^Duration of hospital stay of the patients from South Korea was higher compared with other sites as the majority of them were admitted to ICU.

The study population at the country level, separated by pre-pandemic and pandemic periods, is described in Table 1. Among the study population, patients above 50 years [72.3% (10 124/14 002)] were the predominant than the other age groups [27.6% (3878/14 002)], and there were more males [62.8% (8830/14 055)] than females [37% (5201/14 055)] (Figures S3 and S4). Cases presenting with ‘pneumonia only’ were significantly higher in Bangladesh [89% (1955/2196)] compared with other countries [32% (3791/11 862)] (*P *< 0.0001, OR: 0.058, 95% CI: 0.050–0.067) and ‘sepsis only’ from Italy [61.4% (1469/2392)] compared with other countries [26.5% (3086/11 666] (*P *< 0.0001, OR: 0.226, 95% CI: 0.206–0.248) (Figure S5). Data on COVID-19 testing during the study period were available for 63% (8850/14 058) of the study participants of which 46.7% (4112/8850) were COVID-19 positive, 53.2% (4711/8850) were negative and 0.3% (27/8850) tests were undetermined, with significant differences among countries, e.g. significantly higher COVID-19-negative cases were observed in South Korea than the positive cases, compared with other countries (*P *< 0.0001, OR: 32.960, 95% CI: 25.515–42.579) (Table S5).

### Antimicrobial usage before and during pandemic

A decline (ranging from 9.5% to 20.1%) in the combined prescription of ≥2 antimicrobials during the pandemic was observed in Bangladesh, Italy, Nigeria, Switzerland and Turkey compared with pre-pandemic levels (Table S6). Data from Brazil showed a 43.8% increase in the combined prescription of ≥3 antimicrobials during the pandemic compared with the pre-pandemic period (Table S7).

**Figure 2.**
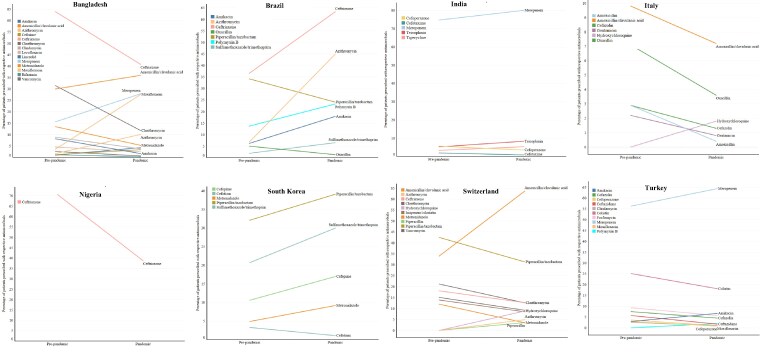
Line graph showing the trends of respective antimicrobial prescriptions from the pre-pandemic to pandemic period at the country level. The ‘x’ axis represents the percentage of patients prescribed with respective antimicrobials. No pre-pandemic data on antimicrobials were available from the Malawi site. Patient-level data could not be accessed from the UK site. Only antimicrobials with significant differences (by chi-square test) in usage between pre-pandemic and pandemic are included in this figure. The antimicrobials with a very low frequency of prescriptions (*n* < 15) are excluded from the figure. Tables S7–S14 demonstrate the differences in usage of all antimicrobials in each country included in this study.

During the study period, 82 different antimicrobials were used across all countries: 39 were from Bangladesh, 37 from Brazil, 38 from India, 41 from Italy, 7 from Malawi, 20 from Nigeria, 44 from South Korea, 42 from Switzerland and 37 from Turkey (Table S8). Tables S9–S16 describe the differences in different classes of antimicrobial usage between the pre-pandemic and pandemic periods across countries. No pre-pandemic data on antimicrobial usage were available from the Malawi site. The antimicrobial prescriptions that significantly increased or decreased during the pandemic are presented in Figure 2.

**Figure 3.**
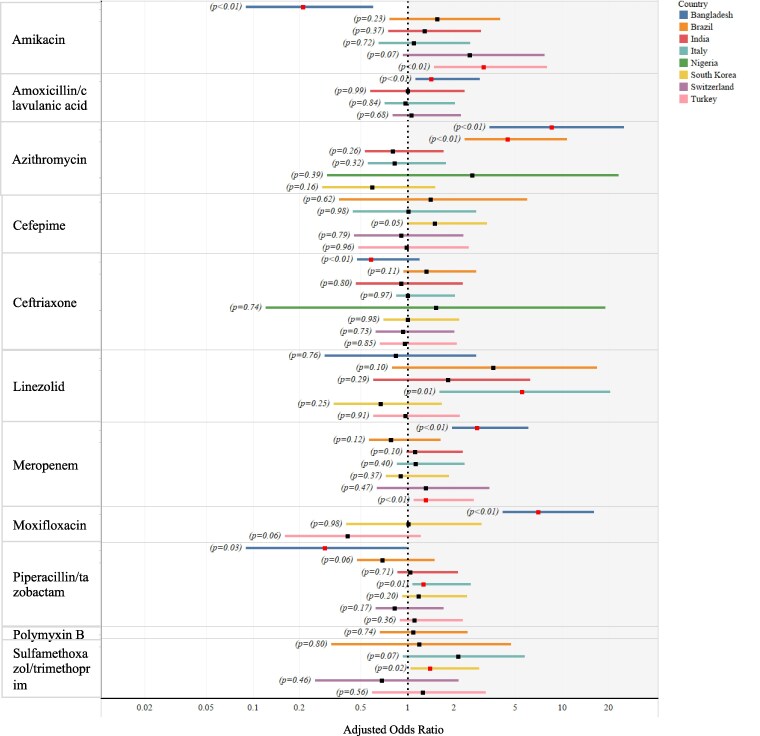
Plot represents the comparison of prescriptions of respective antimicrobials between the pre-pandemic and pandemic periods at the country level. Horizontal bars represent the lower and upper values of a 95% CI. Black square symbols represent the odds ratio, and red square symbols represent tests with significant differences in the prescription of respective antimicrobials between pre-pandemic and pandemic periods. Antimicrobials with <15 prescriptions overall are excluded from the analysis. Statistical analysis was performed using logistic regression. All models are adjusted for age (continuous), sex (male/female/other), admitting ward [ICU/HDU/DCC/COVID specialized (including regular wards for the pre-pandemic period)], comorbidities (binary, yes or no), patient outcome (died/discharged alive) and diagnosis type (sepsis only/pneumonia only/ARDs only/sepsis and pneumonia/sepsis and ARDs/pneumonia and ARDs/sepsis, pneumonia and ARDS). Comparisons of antimicrobial prescriptions between pre-pandemic and pandemic could not be performed using logistic regression if there was a null value either for the pre-pandemic or pandemic period. The difference in amoxicillin/clavulanic acid for Nigeria was not shown in the figure as the adjusted mean difference was zero.

There was an increase in the prescription of amoxicillin-clavulanic acid during the pandemic in Bangladesh (*n* = 4223; *P *= 0.0038; OR: 1.42; 95% CI: 1.12–1.79) with increased DOT (*n* = 617;  *P < *0.0001; GMR: 1.76, 95% CI: 1.49–2.08) with no significant differences in PDD and meropenem (*n* = 4253; *P *< 0.0001; OR: 2.81; 95% CI: 1.94–4.07), with increased PDD (*n* = 549; *P *= 0.0001; GMR: 1.36, 95% CI: 1.17–1.58) and moxifloxacin (*n* = 4251; *P *< 0.0001; OR: 6.99; 95% CI: 4.11–11.87) and with increased DOT (*n* = 449; *P = *0.0099; GMR: 1.71; 95% CI: 1.14–2.56). Ceftriaxone prescription was significantly lower during the pandemic in Bangladesh (*n* = 4251; *P *< 0.0001; OR: 0.58; 95% CI: 0.47–0.72) with decreased PDD in the pandemic compared with pre-pandemic time (*n* = 963; *P *< 0.0001; GMR: 0.85, 95% CI: 0.81–0.89); however, DOT increased by 1.54 days on average (*n* = 822; *P *< 0.0001; GMR: 1.54, 95% CI: 1.34–1.78). Amikacin prescription declined in Bangladesh during the pandemic (*n* = 4085; *P *= 0.0006; OR: 0.21; 95% CI: 0.09 to 0.51) with no significant changes in DOT and PDD (Figures 3, S6 and S7).

There was evidence of significant increase in piperacillin/tazobactam prescriptions in Italy during the pandemic (*n* = 4510; *P *= 0.0046; OR: 1.26; 95% CI: 1.07–1.48) with significantly increased DOT (*n* = 1069; *P *= 0.0336; GMR: 1.12; 95% CI: 1.01–1.25) and PDD (*n* = 1069; *P *= 0.0007; GMR: 1.13; 95% CI: 1.05–1.21) (Figures 3, S6 and S7).

We observed significant increases in meropenem prescriptions in Turkey (*n* = 4012; *P *= 0.0037; OR: 1.31; 95% CI: 1.09–1.58) with reduced DOT (*n* = 849; *P *= 0.0121, GMR: 0.82, 95% CI: 0.71–0.96). Amikacin prescription was higher in Turkey during the pandemic (*n* = 3951; *P *= 0.0026; OR: 3.10; 95% CI: 1.48–6.47) with no significant changes in DOT and PDD (Figures 3, S6 and S7).

Increased probability of using azithromycin in the pandemic was found in all study sites with significantly increased prescriptions in Bangladesh (*n* = 4223; *P *< 0.0001; OR: 8.55; 95% CI: 3.36–21.77) and Brazil (*n* = 3922, *P *< 0.0001; OR: 4.43; 95% CI: 2.33–8.42) and significantly increased DOT by 2.84 days on average in South Korea (*n* = 50; *P *= 0.0009; GMR: 2.84; 95% CI: 1.58–5.11) (Figures 3, S6 and S7).

### Antimicrobial usage in COVID-19-positive versus COVID-19-negative cases

**Figure 4.**
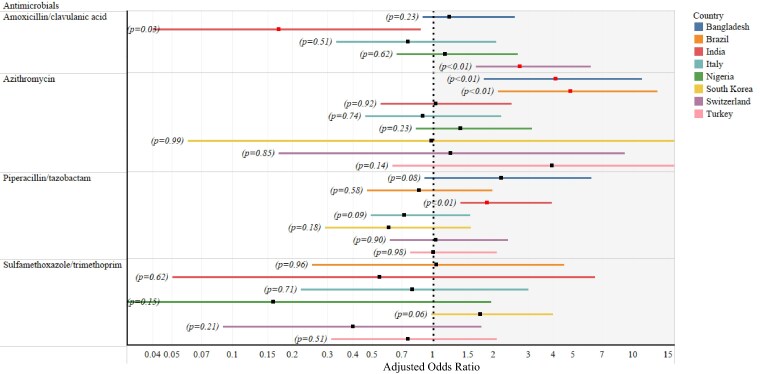
Plot represents the comparison of prescriptions of respective antimicrobials between COVID-19-positive and COVID-19-negative cases at the country level. Horizontal bars represent the lower and upper values of a 95% CI. Black square symbols represent the odds ratio, and red square symbols represent the significant differences in the prescription of respective antimicrobials between COVID-19-positive and COVID-19-negative cases. The antimicrobials with the <15 prescriptions overall are excluded from the analysis. Statistical analysis was performed using logistic regression. All models are adjusted for age (continuous), sex (male/female/other), admitting ward [ICU/HDU/DCC/COVID specialized (including regular wards for the pre-pandemic period)], comorbidities (binary, yes or no), patient outcome (died/discharged alive) and diagnosis type (sepsis only/pneumonia only/ARDS only/sepsis and pneumonia/sepsis and ARDS/pneumonia and ARDS/sepsis, pneumonia and ARDS). Comparisons of antimicrobial prescriptions between COVID-19-positive and COVID-19-negative cases could not be performed using logistic regression if there was a null value either for COVID-19-positive or COVID-19-negative cases.

Subgroup analysis (patients with records of COVID-19 test findings from both pre-pandemic and pandemic periods) demonstrates the higher usage of the following antimicrobials among COVID-19-positive cases compared with negative cases using adjusted logistic regression model: amoxicillin/clavulanic acid in Switzerland (*n* = 1839, *P *= 0.0001; OR: 2.72; 95% CI: 1.64–4.51), azithromycin in Bangladesh (*n* = 1575; *P *= 0.0008; OR: 4.08; 95% CI: 1.80–9.24) and Brazil (*n* = 3455; *P *= 0.0002; OR: 4.84; 95% CI: 2.11–11.08) and piperacillin/tazobactam in India (*n* = 1694; *P *= 0.0001; OR: 1.86; 95% CI: 1.37–2.54) (Figure 4). COVID-19 patients had increased DOT for amoxicillin-clavulanic acid in Nigeria (*n* = 254; *P *= 0.0267; GMR: 1.40; 95% CI: 1.04–1.88) and piperacillin-tazobactam in India (*n* = 280; *P *= 0.0305; GMR: 1.29; 95% CI: 1.02–1.63) (Figure S8). We observed higher PDD for piperacillin-tazobactam in India (*n* = 280; *P *= 0.0433; GMR: 1.13; 95% CI: 1.00–1.27) and Turkey (*n* = 413; *P *= 0.0079; GMR: 1.11; 95% CI: 1.03–1.19) among the COVID-19 cases (Figure S9). Hydroxychloroquine prescriptions were exclusively issued for COVID-19-positive patients and only noted in Italy, Switzerland and Turkey during the first wave of the pandemic.

### Trends in antibiotic usage over the course of the pandemic

**Figure 5.**
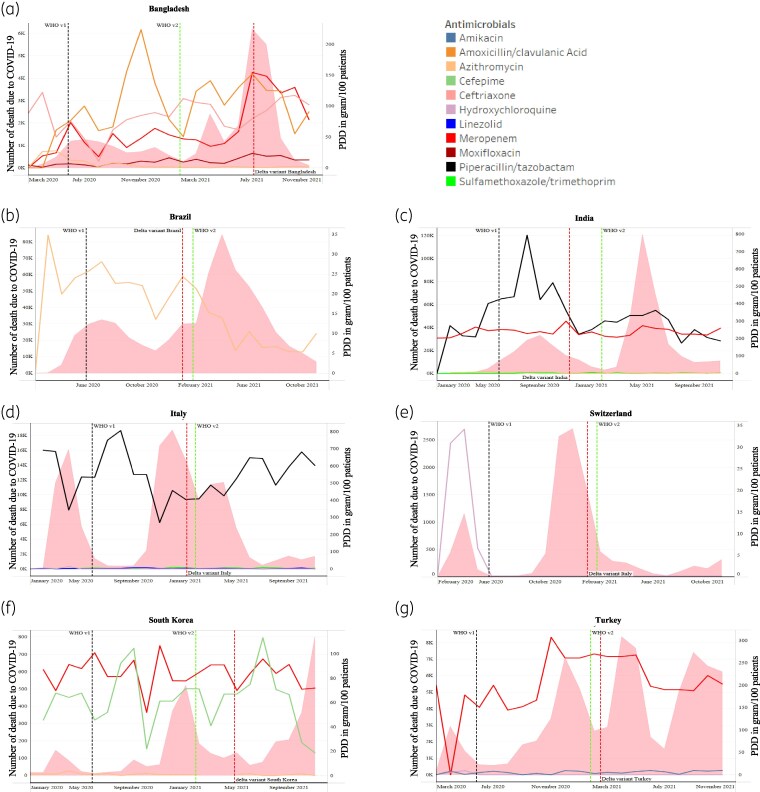
The figures (a-g) represent the mapping of selected antimicrobials at the country level (antimicrobials that were shown to be used significantly during the pandemic using adjusted logistic regression model) with the month-wise incidence of death (represented by pink area plot) and emergence of Delta variant (represented by red dotted line) at the country level and release of WHO guidelines version 1 (represented by black dotted line) and version 2 (represented by green dotted line) on COVID-19 management. Data on incidence of COVID-19 death and COVID-19 vaccination were downloaded from https://data.who.int/dashboards/covid19/data. Data on COVID-19 variants were downloaded from https://ourworldindata.org/grapher/covid-variants-bar. Dates relevant to this figure have been complied with in Table S17. Line plots represent the month-wise PDD of respective antimicrobials in grams per 100 patients.

Figure 5 illustrates monthly antimicrobial usage based on PDD (g)/100 patients against COVID-19 death incidence, Delta variant emergence and when WHO guidelines on COVID patient management were released. ITSA revealed significantly increased meropenem (*n* = 20; *P *< 0.0001; mean change: 109.94, 95% CI: 93.40–126.48 g/100 patients) and moxifloxacin (*n* = 21; *P *= 0.0002; mean change: 9.69, 95% CI: 5.40–13.98 g/100 patients) usage after Delta variant emergence in Bangladesh in July 2021, followed by a downward trend in the post-Delta phase (Figures 5a and S10–S12); however, no significant changes in prescription of ceftriaxone were observed. Azithromycin usage decreased in Bangladesh, Brazil, India and South Korea following WHO guidelines v1 release (Figure 5), which were statistically significant in India (*n* = 17; *P *= 0.0002; mean change: −5.94, 95% CI: −8.38 to −3.49 g/100 patients) and South Korea (*n* = 16; *P *= 0.0274; mean change: −1.82, 95% CI: −2.83 to −1.89 g/100 patients) (Figures S13–S17). Azithromycin usage slightly increased in January 2021 in Brazil and around March 2021 in India (Figures S14 and S15). Post-Delta phase significantly influenced the prescription of more sulfamethoxazole/trimethoprim in India (*n* = 9; *P *= 0.0258; mean change: 5.12, 95% CI: 0.92–9.32 g/100 patients) (Figure S18). A substantial rise in meropenem and amikacin usage was observed in Turkey during November and December 2020, respectively, around the peak of COVID-19 deaths in the second pandemic wave (Figure 5f).

## Discussion

By leveraging individual patient-level data on antibiotic usage patterns and trends in 17 hospitals from 9 countries, this study undertakes granular analysis that enhances statistical power in estimating patient-level antimicrobial consumption during the pandemic compared with the preceding months instead of solely relying on aggregated data, as reported elsewhere.^11,14,15^ By identifying shifts in antibiotic use during the pandemic, the research highlights global differences in prescription practices among varied populations and contexts. These insights shed light on the effectiveness of AMS programmes (AMSP) at the healthcare institutions. While some high-income clinical settings implemented strategic measures to manage antibiotic use during the pandemic, many other participating sites lacked established AMSP (Table S2). The outcomes of this study are valuable for informing future strategies to combat antimicrobial resistance (AMR).

This study included patients exhibiting COVID-like symptoms from critical care units and specialized COVID-19 wards of major hospital in each country, with a significant proportion of participants being over the age of 50, offering insights into antimicrobial prescriptions among moderately to severely ill patients in referral centres. The prevalence of specific diagnoses, such as pneumonia, sepsis and ARDS, varied by sites that might have influenced the type and dosage of antimicrobial therapy prescribed.^17^ Our analysis accounted for these factors, comorbidities, hospital outcome and sex differences, enhancing our understanding of shift in antimicrobial usage during the pandemic.

To address the growing challenge of AMR and maintain antibiotic efficacy, the WHO has set a target for countries to ensure that 60% of antibiotic prescriptions fall within the Access group by 2023.^18^ Our analysis shows a significant increase in the prescribing of Watch group antibiotics such as azithromycin, moxifloxacin, piperacillin/tazobactam and meropenem in hospitals across Bangladesh, Turkey, Italy, Brazil and South Korea during the pandemic. The rapid spread of COVID-19 led to a wide range of clinical outcomes, prompting some regions to include antibiotics in treatment protocols to prevent secondary bacterial infections and for immunomodulation effects.^17–21^ Azithromycin, known for its antibacterial and anti-inflammatory properties, was initially favoured either alone or in combination with hydroxychloroquine. Our study observed an initial surge in azithromycin usage in several countries; however, following WHO guidelines discouraging antibiotic use for COVID-19, prescriptions declined significantly.^22–25^ Other factors such as the provision of the ‘COVID-19 kit’ to the population that included azithromycin, hydroxychloroquine and ivermectin in Brazil aggravated the higher usage of immunomodulatory antimicrobials early in the pandemic.^25^ The capacity of fluoroquinolones to bind with COVID-19 Main Protease (Mpro) might explain moxifloxacin prescriptions for COVID-19.^26^ In addition, meropenem prescriptions in Bangladesh were influenced by national COVID-19 management guidelines (v7) released on 28 May 2020.^27,28^ Reports indicate that around 30% of bacteria from bloodstream infections in Turkey exhibited resistance to third-generation cephalosporins, contributing to the increased use of meropenem.^29^ In Italy, clinicians based antibiotic selections on microbiological evidence, leading to the use of broad-spectrum combinations like piperacillin/tazobactam in response to reduced susceptibility of bacterial strains to third-generation cephalosporins if MICs for piperacillin/tazobactam are ≤4 mg/L, especially for *Escherichia coli* (personal communication with Edoardo Carretto). Overall, the pandemic has had a complex effect on the increased reliance on higher-generation antibiotics.^30^

We analysed monthly aggregated antimicrobial usage data, considering factors such as the emergence of the virulent Delta variant, and the impact of establishment of international treatment guidelines during the first COVID-19 pandemic wave.^31^ The Delta variant caused a significant increase in COVID-19 cases across parts of South Asia, resulting in high mortality rates.^32^ Our study found a significant rise in the prescription of specific antimicrobials in South Asia in the post-Delta phase, notably Watch antibiotics such as meropenem and moxifloxacin in Bangladesh, as well as sulfamethoxazole/trimethoprim in India. Additionally, the influx of critically ill COVID-19 patients in Turkish hospitals during the peak of the second wave might have contributed to increased meropenem usage.^33^

The analysis of antibiotic usage data before and during the pandemic provided insights into the impact of COVID-19 on prescribing practices. Shifts in these patterns may reflect changes in healthcare delivery, including increased telemedicine use, modified diagnostic criteria and altered patient behaviour due to lockdown measures.^17,18^ For instance, our study noted the use of hydroxychloroquine in Italy, Switzerland and Turkey only during the first wave of the pandemic, reflected the rise and fall theories surrounding its effectiveness for treating COVID-19. Initially proposed in March 2020 as a potential treatment, hydroxychloroquine faced criticism and was eventually abandoned following retractions of significant publications from large randomized controlled trials by June and October 2020, culminating in a Cochrane review in 2021.^34^ Although aggregated data from India and Bangladesh indicate a rise in hydroxychloroquine consumption during the pandemic (R. Farzana, S. J. Harbarth, L. Yu, T. R. Walsh and COVID-19/DRI Study Group, unpublished results), our patient-level data found no prescriptions for inpatients in these countries. This suggests that hydroxychloroquine may have been primarily used for pre-exposure prophylaxis among outpatients or in the community rather than for inpatient management.^35^

The prolonged duration of antibiotic prescriptions among COVID-19 patients in countries like Nigeria and India, along with the escalation of dosage in India and Turkey, reflects concerns about treating concurrent bacterial infections or complications related to COVID-19. This situation may stem from a lack of clarity in local clinical guidelines, potentially led to antibiotic misuse or over-prescription.^28–36^

This study has several limitations. We conducted this study during an emergency, which posed challenges to data collection and the adherence to optimal methodological standards across participating sites. Although patient selection guidelines were established, potential selection bias arose from the retrospective case selection using hospital records, which often lacked sufficient clinical details in resource-limited settings. Consequently, we were unable to consistently include 100 patients per month per country. Certain sites encountered challenges in retrieving detailed information from manual records, and data on COVID-19 testing were frequently unavailable during the initial phases of the pandemic, potentially leading to misclassification bias (Table 1). Due to the retrospective nature of the study, our analysis was limited to a 3-month period prior to COVID-19 pandemic.

Our findings provide a comprehensive overview of the variations in antimicrobial usage from baseline across diverse global settings, highlighting significant differences in usage for patients with pneumonia, sepsis and ARDS both before and during the pandemic. The heterogeneity observed among clinical sites is understandable, given the varied contexts of the countries within our network. Several factors likely influenced antimicrobial choice, including the capacity for microbiological testing, the prevalence of bacterial infections, the supply chain dynamics for antimicrobials, cost deferment programmes, COVID-19 management guidelines and overall infection prevention control (IPC) measures.^11–14,19,33^ Understanding how antibiotic usage patterns have changed in response to the pandemic can provide insights into the resilience of healthcare systems and can identify areas where healthcare systems may need attention to maintain appropriate antibiotic-prescribing practices during public health emergencies. Our study highlights the critical need for a robust AMSP as part of pandemic recovery efforts. It advocates for enhancing diagnostic capabilities, implementing effective infection prevention and control measures and refining antimicrobial prescribing policies to address the challenges of AMR during health emergencies.

### Conclusions

This study reinforces the need for AMS during inter-pandemic periods, which is essential for managing future viral outbreaks and addressing the global challenge of AMR.

## Supplementary Material

dlaf037_Supplementary_Data

## Members of the COVID-19/DRI Study Group

Katherine Paulina, Dhiviya Prabaa and Naveen Kumar Devanga Ragupathi from Christian Medical College, Vellore, India. Alpay Azap and Ezgi Gülten from the Department of Infectious Diseases and Clinical Microbiology, Ankara University School of Medicine, Ankara, Türkiye. Elif Ateş, Özlem Azap and Nuran Sarı from the Department of Infectious Diseases and Clinical Microbiology, Başkent University Faculty of Medicine, Ankara, Türkiye. İlkay Karaoğlan, Kübra Koçak and Özlem Mete from the Department of Infectious Diseases and Clinical Microbiology, Gaziantep University Faculty of Medicine, Gaziantep, Türkiye. Emine Coşkun and Murat Kutlu from the Department of Infectious Diseases and Clinical Microbiology, Pamukkale University School of Medicine, Denizli, Türkiye. Şevval Özen Aksakal, Mehtap Aydın and Merve Çağlar Özer from the Department of Infectious Diseases and Clinical Microbiology, Ümraniye Training and Research Hospital, University of Health Sciences, Istanbul, Türkiye. Şirin Menekşe from the Department of Infectious Diseases, Koşuyolu Kartal Heart Training and Research Hospital, İstanbul, Türkiye. Zeynep Ceren Karahan from the Department of Medical Microbiology, Ankara University School of Medicine, Ankara, Türkiye. Begüm Nalça Erdin from the Department of Medical Microbiology, Ümraniye Training and Research Hospital, University of Health Sciences, Istanbul, Türkiye. Cherkaoui Abdessalam, Nadia Colaizzi, Perince Fonton and Cyril Stucki from Infection and Prevention Control, Geneva University Hospitals, Geneva, Switzerland. Riccardo Bianchi, Luca Bruni, Carlo Capatti, Souad Kassem, Francesco Navazio, Michela Paolucci and Benedetta Roatti from IRCCS Azienda Unità Sanitaria Locale, Reggio Emilia, Italy. Khadija Abdulraheem, Tobechi Akujobi, Olanrewaju Falodun and Fortune Fibresima from National Hospital Abuja, Abuja, Nigeria. Abid Anjum Abir, Sabah Arefin, Moumita Deb, Parsa Irin Disha, Farjana Akter Eva, K. Rockybul Hasan, Anantu Islam, Halima Sadia Islam, Kimia Tuj Jannat, Zarin Sultana Liya, Fahmida Afroze Nadia, Afia Anjum Prova, Sadika Rahman, S. M. Rafiqul Islam, Arafat Sabbir, Soumik Talukder and Sultana Jahan Tuly from National Institute of Neurosciences and Hospital, Dhaka, Bangladesh. Lim Jones and Mandy Wootton from Public Health Wales NHS Trust, Public Health Wales, Cardiff, UK.

## Supplementary data

Figures S1–S18 and Tables S1–S17 are available as Supplementary data at *JAC Online*
