# Supplementary material for: The impact of the COVID-19 pandemic on antimicrobial usage: an international patient-level cohort study
Source: JAC Antimicrob Resist. 2025 Mar 26;7(2):dlaf037. doi: 10.1093/jacamr/dlaf037 (PMC11945301; doi:10.1093/jacamr/dlaf037)
Supplement: dlaf037_Supplementary_Data [file dlaf037_supplementary_data.docx]

**Table S1. General information about the sites relevant to this study.**

| **Country** | **Name of the hospital** | **Type of hospital** | **Level of hospital care** | **Total bed numbers** | **Non-COVID ICU^a^** | **Non-COVID HDU^a^** | **COVID ICU^b^** | **COVID HDU^b^** | **COVID wards^b^** | **Microbiology^c^** | **On site COVID-19 testing facility** |
| --- | --- | --- | --- | --- | --- | --- | --- | --- | --- | --- | --- |
| Bangladesh | Dhaka medical College Hospital (DMCH) | Public | Tertiary | 2600 | 20 | 30 | Established 2nd May 2020 with 10 beds; this increased to 20 on 1st April 2021 | Established 18th May 2020 with 24 beds; this decreased to 11 on 27th May 2021 | Established 2nd May 2020 with 208 beds, this increased to 594 on 16th May 2020 | On site microbiology in place, not accredited | COVID-19 testing facility available (PCR using Sansure kit/ABI Biosystems 7500) |
|  | Shaheed Suhrawardy Medical College Hospital (ShMCH) | Public | Tertiary | 850 | 10 | 13 | Dedicated ICU established on 3rd April 2020 with 10 beds | Dedicated HDU established on 31st July 2020 with 13 beds | Specialized COVID-19 ward established on 3rd April 2020 with 200 beds | On site microbiology in place, not accredited | COVID-19 testing facility available (PCR using Sansure kit/ABI Biosystems 7500) |
|  | National Institute of Neurosciences and Hospital (NINS) | Public | Tertiary | 450 | 12 | 9 | No | No | Specialized COVID-19 ward established on 12th May 2020 with 16 beds, increased to 100 on 16th May 2020 | On site microbiology in place, not accredited | COVID-19 testing facility available (rapid antigen test using Standard TM Q Covid 19 Ag test [SD BIOSENSOR]) |
| Brazil | Federal University of São Paulo (UNIFESP) | Public | Tertiary | 600 | 48 | 10 | Dedicated ICU established on 1st April 2020 with 35 beds; increased to 44 on 1st June 2020; reduced to 35 on 1st September 2020; reduced to 26 on 1st November 2020; increased to 35 on 1st December 2020; increased to 44 on 15th April 2021; reduced to 26 on 1st June 2021 and to 17 on 06th October 2021; Dedicated ICU closed on November 2021 | No | Dedicated COVID wards on 1st April 2020 38 beds; increased to 50 on 1st May 2020; reduced to 40 on 1st July 2020; reduced to 20 on 1st September 2020 | On site microbiology in place, accredited by Clinical Laboratory Accreditation Program of Brazilian Society of Clinical Pathology (since 2013) | COVID-19 testing facility available (PCR using GeneFinder® COVID-19 Plus) |
| India | Christian Medical College (CMC) | Private-non-profit | Tertiary | 2200 | NA | NA | Dedicated ICU established on 26th August with 112 beds; increased to 1054 on 16th September 2022. | Dedicated HDU established on 20th June 2020 with 33 beds. | Dedicated ward established January 2020. Beds varied by time point, 50 beds currently in operation from August 2022. In the peak pandemic period from March to April 2022, the number of beds was up to 85. | On site microbiology is available, accredited by National Accreditation Board for Testing and Calibration Laboratories, India (15189-2012) | COVID-19 testing facility is available (PCR using RealStar® SARS-CoV-2 RT-PCR Kit 1.0) |
| Italy | Arcispedale Santa Maria Nuova - AUSL Reggio Emilia (ASMN) | Public | Tertiary | 856 | 12 | 32 | Dedicated ICU established on 10th March 2020 with 40 beds; reduced to 5 by 8th June 2021 | Dedicated HDU established on 23rd March 2020 with 16 beds; decreased to 10 by 11th December 2021 | Specialized COVID-19 ward established on 20th March 2020 with 200 beds, reduced to 23 by 10th Aug 2021 | On site microbiology is available, accredited by Regione Emilia Romagna | COVID-19 testing facility available (PCR using Cepheid, Seegene, Roche, Elitech, Diasorin; serology using Diasorin, Vircell; rapid antigen test using Boditech Med Inc [EU distr., Menarini]) |
| South Korea | Yonsei University Health System (YUHS) | Private-non-profit | Tertiary | 2600 | 226 | No | No | No | Dedicated ward established January 2020. The number of COVID beds varies by time point, 50 beds in operation from August 2022. In the peak pandemic period, the number was up to 85 from March to April 2022. | On site microbiology is available, accredited by College of American Pathologists and Joint Commission International | COVID-19 testing facility is available (PCR using Seegene, GeneXpert, Roche) |
| Malawi | Queen Elizabeth Central Hospital (QECH) | Public | Tertiary | 1350 | 4 | No | No | Dedicated HDU established 23rd March 2020 with 16 beds; decreased to 10 by 11th December 2021 | Specialized COVID-19 ward established on 20th March 2020 with 200 beds, bed number reduced to 23 by 10th Aug 2021 | On site microbiology is available, accredited by Southern African Development Community Accreditation Service | COVID-19 testing facility is available (PCR using Viia7 machine, Quantbio qScript® One-Step qRT-PCR Kit, Low ROX™ SARS-CoV-2 primer/probe mix) |
| Nigeria | National Hospital Abuja (NHA) | Public | Tertiary | 439 | 40 | No | Dedicated ICU established on 20th Sep 2021 with 8 beds | No | No | On site microbiology is available, accredited by West African College of Physicians and National Post graduate Medical College of Nigeria | COVID-19 testing facility available (PCR Quantstudio 5, MIC, GeneXpert. using Liferiver kit) |
| Switzerland | Geneva University Hospitals (HUG) | Public | Tertiary | 2505 | 32 | 40 | Dedicated ICU established on 1st April 2020 with 12beds; reduced to 2 by 1st January 2021 | Dedicated HDU established on 1st August 2020 with 11 beds increased to 44 on 1st November 2021 | Specialized COVID-19 ward established on 1st August 2020 with 28 beds, increased to 227 on 1st December 2020 | On site microbiology is available, accredited by International Organization for Standardization (ISO 15189 SMTS 0032) | COVID-19 testing facility is available (PCR using Roche COBAS 6800 / Genexpert; serology using Euroimmun SARS IgG et IgA) |
| Turkey | Koc University Hospital (KUH) | Private-non-profit | Tertiary | 371 | 55 | No | No | No | Specialized COVID-19 ward established on 11th March 2020 with 34 beds, closed on 1st April 2022 | On site microbiology available, accredited by National accreditation and external quality control | COVID-19 testing facility is available (PCR using Qiagen [Qiaamp]) |
|  | Ümraniye Training and Research Hospital (UH) | Public | Tertiary | 856 | 70 | No | No | No | Specialized COVID-19 ward established 20th March 2020 with 50 beds, closed on 10th July 2022 | On site microbiology is available, not accredited | COVID-19 testing facility is available (PCR against targeted genes, ORF1 and N gene) |
|  | Ankara University İbni Sina Hospital (AUH) | Public | Tertiary | 1094 | 75 | No | Dedicated ICU established on 17th March 2020 with 10 beds; increased to 18 by 1st June 2021 | No | Specialized COVID-19 ward established on 17th March 2020 with 45 beds, increased to 120 by 1^st^ September 2021, then again reduced to 45 by 1st December 2021 | On site microbiology is available, accredited by National accreditation and external quality control | COVID-19 testing facility is available (PCR using Rotor-Gene Q MDx 5Plex and Coronogen, Coronex, RTA, Bioksen) |
|  | Baskent Universty (BUH) | Private-profit | Tertiary | 368 | 78 | No | Dedicated ICU established on 22nd March 2020 with 9 beds | No | Specialized COVID-19 ward established on 22nd March 2020 with 37 beds, reduced to 12 by 8th March 2021 | On site microbiology is available, accredited by International Organization for Standardization (TSE-ISO-EN- 9000, TS EN ISO 9001) | COVID-19 testing facility is available (PCR using Rotor-Gene Q MDx 5Plex and Rotor-Gene Q, rRT PCR) |
|  | Gaziantep University Hospital (GUH) | Public | Tertiary | 995 | 20 | No | Dedicated ICU established on 14th March 2020 with 16 beds | No | Specialized COVID-19 ward established on 14th March 2020 with 30 beds, reduced to 16 by 12^th^ January 2021 | On site microbiology is available, accredited by International Organization for Standardization (15189) | COVID-19 testing facility is available (PCR using Roche) |
|  | Pamukkale University (PUH) | Public | Tertiary | 888 | 102 | No | Dedicated ICU established on 10th March 2020 with 8 beds ; reduced to 5 by 4th November 2020 | No | Specialized COVID-19 ward established on 10th March 2020 with 30 beds, increased to 68 by 14th November 2020 | On site microbiology is available, not accredited | COVID-19 testing facility is available |
|  | Kartal Kosuyolu Training Hospital (KKH) | Public | Tertiary | 465 | 127 | No | Dedicated ICU established on 1st April 2020 with 12 beds | No | No | On site microbiology is available, accredited by National accreditation and external quality control | No COVID-19 testing facility is available |

^a^Bed number; ^b^establishment and changes in the capacity of ICU, HDU, and specialized wards at each clinical site over time; ^c^detailed microbiology capacity at each clinical site is stated in Table S2. NA, data not available.

**Table S2. Microbiology capacity and status of Antibiotic Stewardship Program (ASP) of the clinical sites included in this study.**

|  | **Bangladesh** | | | **Brazil** | **India** | **Italy** | **South Korea** | **Malawi** | **Nigeria** | **Switzerland** | **Turkey** | | | | | | |
| --- | --- | --- | --- | --- | --- | --- | --- | --- | --- | --- | --- | --- | --- | --- | --- | --- | --- |
|  | DMCH | ShMCH | NINS | UNIFESP | CMC | ASMN | YUHS | QECH | NHA | HUG | KUH | UH | AUH | BUH | GUH | PUH | KKH |
| On-site microbiology | ✅ | ✅ | ✅ | ✅ | ✅ | ✅ | ✅ | ✅ | ✅ | ✅ | ✅ | ✅ | ✅ | ✅ | ✅ | ✅ | ✅ |
| Laboratory accreditation is in place | ❌ | ❌ | ❌ | ✅ | ✅ | ✅ | ✅ | ✅ | ✅ | ✅ | ✅ | ✅ | ✅ | ✅ | ✅ | ❌ | ✅ |
| Automated blood culture facility is available | ❌ | ❌ | ✅ | ✅ | ✅ | ✅ | ✅ | ✅ | ✅ | ✅ | ✅ | ✅ | ✅ | ✅ | ✅ | ✅ | ✅ |
| Manual blood culture facility is available | ✅ | ✅ | ✅ | ❌ | ❌ | ❌ | ❌ | ❌ | ❌ | ❌ | ❌ | ❌ | ❌ | ❌ | ❌ | ❌ | ❌ |
| Bacterial identification by biochemical tests | ✅ | ✅ | ✅ | ❌ | ❌ | ❌ | ❌ | ✅ | ✅ | ❌ | ❌ | ❌ | ❌ | ❌ | ❌ | ❌ | ❌ |
| Bacterial identification by MALDI-TOF MS | ❌ | ❌ | ❌ | ✅ | ✅ | ✅ | ✅ | ❌ | ❌ | ✅ | ❌ | ✅ | ✅ | ❌ | ❌ | ❌ | ❌ |
| Bacterial identification and AST by VITEK-2 | ❌ | ❌ | ❌ | ✅ | ✅ | ❌ | ✅ | ❌ | ✅ | ❌ | ✅ | ✅ | ✅ | ✅ | ✅ | ✅ | ✅ |
| Bacterial identification and AST by BD Phoenix™ | ❌ | ❌ | ❌ | ✅ | ✅ | ✅ | ❌ | ❌ | ❌ | ❌ | ❌ | ❌ | ❌ | ✅ | ❌ | ❌ | ❌ |
| AST by disk diffusion | ✅ | ✅ | ✅ | ✅ | ✅ | ✅ | ✅ | ✅ | ✅ | ✅ | ✅ | ✅ | ✅ | ✅ | ✅ | ✅ | ✅ |
| AST by broth microdilution (not automated) | ❌ | ❌ | ❌ | ✅ | ✅ | ✅ | ❌ | ❌ | ❌ | ✅ | ❌ | ✅ | ❌ | ❌ | ❌ | ❌ | ❌ |
| AST by E-test | ❌ | ❌ | ❌ | ❌ | ✅ | ✅ | ❌ | ❌ | ❌ | ❌ | ✅ | ✅ | ❌ | ❌ | ❌ | ❌ | ❌ |
| CLSI guideline | ✅ | ✅ | ✅ | ❌ | ✅ | ❌ | ✅ | ❌ | ❌ | ❌ | ❌ | ❌ | ❌ | ❌ | ❌ | ❌ | ❌ |
| EUCAST guideline | ❌ | ❌ | ❌ | ✅ | ❌ | ✅ | ❌ | ✅ | ✅ | ✅ | ✅ | ✅ | ✅ | ✅ | ✅ | ✅ | ✅ |
| ASP including treatment guidelines (including review dates) in place | ❌ | ❌ | ❌ | ✅ | ✅ | ✅ | ✅ | ✅ | ❌ | ✅ | ✅ | ❌ | ✅ | ❌ | ✅ | ✅ | ✅ |
| Any changes/modifications to ASP during COVID-19 pandemic | NA | NA | NA | No | No | Yes, the ASP programmes were adapted to the clinical and epidemiological variations entailed by Covid-19. There was a total increase of infectious consultations for Covid and non-covid wards.  Internal guidelines concerning fungal infection managements and screening programs for aspergillosis associated with Covid for ICU were published.  In January 2021, the hospital faced wide outbreak due to a *Klebsiella pneumoniae* clone which was CTX-M positive, CMY-2 positive, with significant decrease of susceptibility to carbapenems (mostly, meropenem and ertapenem). An empirical antibiotic scheme was therefore adopted during this outbreak to cover isolates with this peculiar form of antimicrobial resistance. | No | No | No | Yes, multiple changes related to CoVID-19 treatment guidelines and re-introduction of institutional availability of procalcitonin testing. Multiple monthly changes to treatment guidelines from April 2020 to Novembre 2021. | No | No | No | No | No | No | No |
| Any change in patients' management policy due to stock out of antibiotics during COVID-19 pandemic | No | No | No | Yes, A contingency plan was carried out aimed at the treatment of Gram-negative bacteria resistant to carbapenems due to the lack of Polymyxin B. | No | No | No | No | No | Yes, temporary shortages of IV amoxicillin unrelated to the CoVID-19 pandemic. Exact dates of changes and shortages unknown. | No | No | No | No | No | No | No |
| Any change in patients' management policy such as prophylactic usage of antimicrobials during COVID-19 pandemic | Yes | Yes | Yes, usage of Azithromycin as prophylaxis from first week of April 2021 to last week of October 2021 | No | No | Yes, the decision not to introduce prophylactic antibiotics for patients on ventilator support was a strategic move aimed at reducing overall antibiotic consumption. This approach stands in contrast to trends observed in other regional hospitals, which have seen an increase in antibiotic usage. Specifically, from March 2020 to November 2021, there was a noticeable rise in the use of WATCH group antibiotics, such as carbapenems and piperacillin/tazobactam, while the use of macrolides was decreased. | Yes | No | No | No general changes in antibiotic prophylaxis guidelines. However, introduction of a computerized decision support system in summer 2021 for the operating room area | No | No | No | No | Yes | No | No |

The symbol ‘✅’ indicates ‘presence’, ‘❌’ indicates ‘absence, NA, not applicable.

**Table S3. Changes in IPC policy during the pandemic in the clinical sites included in this study.**

|  | **Bangladesh** | | | **Brazil** | **India** | **Italy** | **South Korea** | **Malawi** | **Nigeria** | **Switzerland** | **Turkey** | | | | | | |
| --- | --- | --- | --- | --- | --- | --- | --- | --- | --- | --- | --- | --- | --- | --- | --- | --- | --- |
|  | DMCH | ShMCH | NINS | UNIFESP | CMC | ASMN | YUHS | QECH | NHA | HUG | KUH | UH | AUH | BUH | GUH | PUH | KKH |
| Usage of disposable apron, gloves, eye and face protection together by HCW for providing care to any patient | ❌ | ❌ | ❌ | ✅ | ✅ | ✅ | ✅ | ✅ | ✅ | ✅ | ❌ | ✅ | ✅ | ❌ | ❌ | ✅ | ✅ |
| Usage of disposable apron, gloves, eye and face protection together by HCW for providing care to COVID patients | ✅ | ✅ | ✅ | ✅ | ✅ | ✅ | ✅ | ✅ | ✅ | ✅ | ❌ | ✅ | ✅ | ✅ | ❌ | ✅ | ✅ |
| Establishment of COVID isolation unit | ✅ | ✅ | ✅ | ✅ | ✅ | ✅ | ✅ | ✅ | ✅ | ✅ | ✅ | ❌ | ✅ | ✅ | ✅ | ✅ | ✅ |
| COVID-19 testing for any new admissions during the peak | ❌ | ❌ | ❌ | ✅ | ✅ | ✅ | ✅ | ✅ | ❌ | ✅ | ❌ | ✅ | ✅ | ✅ | ❌ | ✅ | ✅ |
| COVID-19 testing for any new admissions with suspected COVID-19 during the peak | ✅ | ✅ | ✅ | ✅ | ✅ | ✅ | ✅ | ✅ | ✅ | ✅ | ✅ | ✅ | ✅ | ✅ | ✅ | ✅ | ✅ |
| Periodic COVID-19 checks for any hospitalized patient during the peak | ❌ | ❌ | ❌ | ❌ | ✅ | ✅ | ✅ | ✅ | ❌ | ✅ | ❌ | ❌ | ❌ | ❌ | ❌ | ❌ | ❌ |
| Periodic COVID-19 testing for the hospitalized patients with suspected COVID-19 during the peak | ✅ | ✅ | ✅ | ✅ | ✅ | ✅ | ✅ | ✅ | ✅ | ✅ | ✅ | ❌ | ✅ | ❌ | ✅ | ✅ | ✅ |
| Usage of disinfectants at the entrance of the hospitals, lifts | ✅ | ✅ | ✅ | ✅ | ✅ | ✅ | ✅ | ✅ | ✅ | ✅ | ✅ | ❌ | ✅ | ✅ | ✅ | ✅ | ✅ |
| Control or limitation of visitor access | ✅ | ✅ | ✅ | ✅ | ✅ | ✅ | ✅ | ✅ | ✅ | ✅ | ✅ | ✅ | ✅ | ✅ | ✅ | ✅ | ✅ |
| Implementation of social distancing | ✅ | ✅ | ✅ | ✅ | ✅ | ✅ | ✅ | ✅ | ✅ | ✅ | ✅ | ✅ | ✅ | ✅ | ✅ | ✅ | ✅ |

The symbol ‘✅’ indicates ‘yes’ and ‘❌’ indicates ‘no’.

**Table S4. References of ethical approval.**

| **Country** | **Reference number for ethical approval** |
| --- | --- |
| United Kingdom | IRAS project ID: 299985 |
| Bangladesh | BMRC/NREC/2019-2022/279 |
| Brazil | 57642022.0.0000.5505 |
| India | 2021-8083 |
| Italy | 788/2021/OSS/AUSLRE |
| South Korea | 4-2021-1644 |
| Malawi | NHSRC, 20/02/2518 and 19/08/2246; LSTM REC, 20/026 and 19/017 |
| Nigeria | NHA/EC/061/2021 |
| Switzerland | 2021-01820 |
| Turkey | 2021.322.IRBI.148 |

**Table S5. Differences in COVID-19 test positivity among the study population at the country level (n=8823).**

| **COVID-19 test findings** | **Bangladesh (n=684)** | **Other countries (n=8139)** | ***p* value** | **OR** | **95% CI** |
| --- | --- | --- | --- | --- | --- |
| COVID-19 positive | 470 (68.7) | 3642 (44.7) | <0.0001 | 0.369 | 0.312-0.436 |
| COVID-19 negative | 214 (31.3) | 4497 (55.3) |  |  |  |
| **COVID-19 test findings** | **Brazil (n=1102)** | **Other countries (n=7721)** | ***p* value** | **OR** | **95% CI** |
| COVID-19 positive | 1025 (93) | 3087 (40.0) | <0.0001 | 0.050 | 0.040-0.063 |
| COVID-19 negative | 77 (7) | 4634 (60.0) |  |  |  |
| **COVID-19 test findings** | **India (n=999)** | **Other countries (n=7824)** | ***p* value** | **OR** | **95% CI** |
| COVID-19 positive | 430 (43.0) | 3682 (47.1) | 0.0165 | 1.176 | 1.030-1.343 |
| COVID-19 negative | 569 (57.0) | 4142 (52.9) |  |  |  |
| **COVID-19 test findings** | **Italy (n=1733)** | **Other countries (n=7090)** | ***p* value** | **OR** | **95% CI** |
| COVID-19 positive | 432 (24.9) | 3680 (51.9) | <0.0001 | 3.250 | 2.887-3.658 |
| COVID-19 negative | 1301 (75.1) | 3410 (48.1) |  |  |  |
| **COVID-19 test findings** | **Malawi (n=405)** | **Other countries (n=8418)** | ***p* value** | **OR** | **95% CI** |
| COVID-19 positive | 321 (79.3) | 3791 (45.0) | <0.0001 | 0.214 | 0.168-0.274 |
| COVID-19 negative | 84 (20.7) | 4627 (55.0) |  |  |  |
| **COVID-19 test findings** | **Nigeria (n=399)** | **Other countries (n=8424)** | ***p* value** | **OR** | **95% CI** |
| COVID-19 positive | 358 (89.7) | 3754 (44.6) | <0.0001 | 0.092 | 0.066-0.128 |
| COVID-19 negative | 41 (10.3) | 4670 (55.4) |  |  |  |
| **COVID-19 test findings** | **South Korea (n=1660)** | **Other countries (n=7163)** | ***p* value** | **OR** | **95% CI** |
| COVID-19 positive | 63 (3.8) | 4049 (56.5) | <0.0001 | 32.960 | 25.515-42.579 |
| COVID-19 negative | 1597 (96.2) | 3114 (43.5) |  |  |  |
| **COVID-19 test findings** | **Switzerland (n=852)** | **Other countries (n=7971)** | ***p* value** | **OR** | **95% CI** |
| COVID-19 positive | 767 (90.0) | 3345 (42.0) | <0.0001 | 0.080 | 0.064-0.101 |
| COVID-19 negative | 85 (10.0) | 4626 (58.0) |  |  |  |
| **COVID-19 test findings** | **Turkey (n=989)** | **Other countries (n=7834)** | ***p* value** | **OR** | **95% CI** |
| COVID-19 positive | 246 (24.9) | 3866 (49.3) | <0.0001 | 2.943 | 2.531-3.422 |
| COVID-19 negative | 743 (75.1) | 3968 (50.7) |  |  |  |

Among the study population, COVID-19 test findings were available for 8,850 cases. This analysis did not include the cases where the findings were undetermined (n=27). Chi-squared/Fisher’s exact tests (unadjusted) were performed to assess the COVID-19 positivity (binary for yes/no against COVID-19-positive/COVID-19-negative) among countries (yes/no against the country under investigation vs combined data from other countries). The Odds Ratio shows the odds of having a negative COVID test, comparing the country under investigation and all of the other countries during the pandemic periods.

**Table S6. Differences in the prescription of a combination of two or more antimicrobial therapies between pre-pandemic and pandemic periods.**

| **Country** | **Pre-pandemic** | **Pandemic** | **Changes in percentage (%)*** | ***p* value** | **OR** | **95% CI** |
| --- | --- | --- | --- | --- | --- | --- |
| Bangladesh | 62.7% (261/416) | 50.1% (891/1780) | 20.1 | <0.0001 | 0.595 | 0.478-0.741 |
| Brazil | 77.6% (125/161) | 82.1% (929/1131) | 5.8 | 0.1682 | 1.325 | 0.887-1.977 |
| India | 41.1% (312/760) | 42.2% (775/1837) | 2.7 | 0.5935 | 1.048 | 0.883-1.244 |
| Italy | 43.7% (214/490) | 37.4% (711/1902) | 14.4 | 0.0108 | 0.770 | 0.630-0.942 |
| Nigeria | 94.1% (16/17) | 85.7% (401/468) | 8.9 | 0.3252 | 0.374 | 0.049-2.868 |
| South Korea | 67.5% (166/246) | 71.8% (1285/1789) | 6.4 | 0.1575 | 1.229 | 0.923-1.635 |
| Switzerland | 58.3% (151/259) | 47.6% (444/932) | 18.3 | 0.0024 | 0.651 | 0.493-0.860 |
| Turkey | 81.4% (227/279) | 73.7% (867/1176) | 9.5 | 0.0079 | 0.643 | 0.463-0.892 |

*Cells highlighted in ‘yellow’ indicate a decreased percentage of antimicrobial usage during the pandemic and cells highlighted in ‘blue’ indicate an increased percentage of usage. Chi-squared/Fisher’s exact tests (unadjusted) were performed to assess the usage of combined antimicrobials (binary for yes/no against usage of combination therapy) among countries (yes/no against the usage in the country under investigation vs combined data from other countries).

**Table S7. Differences in the prescription of a combination of three or more antimicrobial therapies between pre-pandemic and pandemic periods.**

| **Country** | **Pre-pandemic** | **Pandemic** | **Changes in percentage (%)*** | ***p* value** | **OR** | **95% CI** |
| --- | --- | --- | --- | --- | --- | --- |
| Bangladesh | 20.9% (87/416) | 18.5% (330/1780) | 11.4 | 0.2663 | 0.861 | 0.660-1.122 |
| Brazil | 31.7% (51/161) | 45.6% (516/1131) | 43.8 | 0.0008 | 1.810 | 1.273-2.573 |
| India | 5% (38/760) | 3% (56/1837) | 40.0 | 0.0154 | 0.597 | 0.392-0.910 |
| Italy | 16.5% (81/490) | 10.4% (197/1902) | 36.9 | 0.0001 | 0.583 | 0.441-0.772 |
| Nigeria | 29.4% (5/17) | 12.2% (57/468) | 58.5 | 0.0366 | 0.333 | 0.113-0.980 |
| South Korea | 41.9% (103/246) | 47.7% (853/1789) | 13.8 | 0.0869 | 1.265 | 0.966-1.657 |
| Switzerland | 19.7% (51/259) | 18.7% (174/932) | 5.1 | 0.7102 | 0.936 | 0.661-1.326 |
| Turkey | 44.1% (123/279) | 43.6% (513/1176) | 1.1 | 0.8884 | 0.981 | 0.754-1.276 |

*Cells highlighted in ‘yellow’ indicate a decreased percentage of antimicrobial usage during the pandemic and cells highlighted in ‘blue’ indicate an increased percentage of usage. Chi-squared/Fisher' exact tests were performed to assess the usage of combined antimicrobials (binary for yes/no against usage of combination therapy) among countries (yes/no against the usage in the country under investigation vs combined data from other countries).

**Table S8. The variations and similarities in the types of antimicrobials prescribed among study participants across different countries.**

| **Class** | **AWaRe classes** | **Antimicrobials** | **Listed under WHO Essential Medicine** | **Bangladesh** | **Brazil** | **India** | **Italy** | **Malawi** | **Nigeria** | **South Korea** | **Switzerland** | **Turkey** |
| --- | --- | --- | --- | --- | --- | --- | --- | --- | --- | --- | --- | --- |
| Penicillins | Access | Amoxicillin | Yes |  |  |  |  |  |  |  |  |  |
| Penicillins | Access | Ampicillin | Yes |  |  |  |  |  |  |  |  |  |
| Penicillins | Access | Benzylpenicillin | Yes |  |  |  |  |  |  |  |  |  |
| Penicillins | Access | Cloxacillin | Yes |  |  |  |  |  |  |  |  |  |
| Penicillins | Access | Dicloxacillin | No |  |  |  |  |  |  |  |  |  |
| Penicillins | Access | Flucloxacillin | No |  |  |  |  |  |  |  |  |  |
| Penicillins | Access | Oxacillin | No |  |  |  |  |  |  |  |  |  |
| Penicillins | Access | Phenoxymethylpenicillin | Yes |  |  |  |  |  |  |  |  |  |
| Beta lactam - beta lactamase inhibitor | Access | Amoxicillin/clavulanicAcid | Yes |  |  |  |  |  |  |  |  |  |
| Beta lactam - beta lactamase inhibitor | Access | Ampicillin/sulbactam | No |  |  |  |  |  |  |  |  |  |
| First-generation cephalosporins | Access | Cefalotin | No |  |  |  |  |  |  |  |  |  |
| First-generation cephalosporins | Access | Cefazolin | Yes |  |  |  |  |  |  |  |  |  |
| First-generation cephalosporins | Access | Cefradine | No |  |  |  |  |  |  |  |  |  |
| First-generation cephalosporins | Access | Cefroxadine | No |  |  |  |  |  |  |  |  |  |
| Aminoglycosides | Access | Amikacin | Yes |  |  |  |  |  |  |  |  |  |
| Aminoglycosides | Access | Gentamicin | Yes |  |  |  |  |  |  |  |  |  |
| Tetracyclines | Access | Doxycycline | Yes |  |  |  |  |  |  |  |  |  |
| Trimethoprim - sulfonamide combinations | Access | Sulfadiazine/trimethoprim | No |  |  |  |  |  |  |  |  |  |
| Trimethoprim - sulfonamide combinations | Access | Sulfamethizole/trimethoprim | No |  |  |  |  |  |  |  |  |  |
| Trimethoprim - sulfonamide combinations | Access | Sulfamethoxazole/trimethoprim | Yes |  |  |  |  |  |  |  |  |  |
| Trimethoprim - sulfonamide combinations | Access | Sulfametrole/trimethoprim | No |  |  |  |  |  |  |  |  |  |
| Trimethoprim - sulfonamide combinations | Access | Sulfamoxole/trimethoprim | No |  |  |  |  |  |  |  |  |  |
| Amphenicols | Access | Chloramphenicol | Yes |  |  |  |  |  |  |  |  |  |
| Imidazoles | Access | Metronidazole | Yes |  |  |  |  |  |  |  |  |  |
| Lincosamides | Access | Clindamycin | Yes |  |  |  |  |  |  |  |  |  |
| Nitrofurantoin | Access | Nitrofurantoin | No |  |  |  |  |  |  |  |  |  |
| Penicillins | Watch | Azlocillin | No |  |  |  |  |  |  |  |  |  |
| Penicillins | Watch | Piperacillin | No |  |  |  |  |  |  |  |  |  |
| Penicillins | Watch | Sulbenicillin | No |  |  |  |  |  |  |  |  |  |
| Beta lactam - beta lactamase inhibitor (anti-pseudomonal) | Watch | Piperacillin/tazobactam | Yes |  |  |  |  |  |  |  |  |  |
| Second-generation cephalosporins | Watch | Cefaclor | No |  |  |  |  |  |  |  |  |  |
| Second-generation cephalosporins | Watch | Cefbuperazone | No |  |  |  |  |  |  |  |  |  |
| Second-generation cephalosporins | Watch | Cefotetan | No |  |  |  |  |  |  |  |  |  |
| Second-generation cephalosporins | Watch | Cefotiam | No |  |  |  |  |  |  |  |  |  |
| Second-generation cephalosporins | Watch | Cefoxitin | No |  |  |  |  |  |  |  |  |  |
| Second-generation cephalosporins | Watch | Cefprozil | No |  |  |  |  |  |  |  |  |  |
| Second-generation cephalosporins | Watch | Cefuroxime | Yes |  |  |  |  |  |  |  |  |  |
| Second-generation cephalosporins | Watch | Flomoxef | No |  |  |  |  |  |  |  |  |  |
| Third-generation cephalosporins | Watch | Cefixime | Yes |  |  |  |  |  |  |  |  |  |
| Third-generation cephalosporins | Watch | Cefodizime | No |  |  |  |  |  |  |  |  |  |
| Third-generation cephalosporins | Watch | Cefoperazone | No |  |  |  |  |  |  |  |  |  |
| Third-generation cephalosporins | Watch | Cefotaxime | Yes |  |  |  |  |  |  |  |  |  |
| Third-generation cephalosporins | Watch | Cefpiramide | No |  |  |  |  |  |  |  |  |  |
| Third-generation cephalosporins | Watch | Cefpodoxime | No |  |  |  |  |  |  |  |  |  |
| Third-generation cephalosporins | Watch | Ceftazidime | Yes |  |  |  |  |  |  |  |  |  |
| Third-generation cephalosporins | Watch | Ceftibuten | No |  |  |  |  |  |  |  |  |  |
| Third-generation cephalosporins | Watch | Ceftriaxone | Yes |  |  |  |  |  |  |  |  |  |
| Fourth-generation cephalosporins | Watch | Cefepime | Yes |  |  |  |  |  |  |  |  |  |
| Carbapenems | Watch | Doripenem | No |  |  |  |  |  |  |  |  |  |
| Carbapenems | Watch | Ertapenem | No |  |  |  |  |  |  |  |  |  |
| Carbapenems | Watch | Imipenem/cilastatin | Yes |  |  |  |  |  |  |  |  |  |
| Carbapenems | Watch | Meropenem | Yes |  |  |  |  |  |  |  |  |  |
| Aminoglycosides | Watch | Arbekacin | No |  |  |  |  |  |  |  |  |  |
| Aminoglycosides | Watch | Isepamicin | No |  |  |  |  |  |  |  |  |  |
| Aminoglycosides | Watch | Netilmicin | Yes |  |  |  |  |  |  |  |  |  |
| Aminoglycosides | Watch | Tobramycin | Yes |  |  |  |  |  |  |  |  |  |
| Fluoroquinolones | Watch | Gemifloxacin | No |  |  |  |  |  |  |  |  |  |
| Fluoroquinolones | Watch | Ciprofloxacin | Yes |  |  |  |  |  |  |  |  |  |
| Fluoroquinolones | Watch | Levofloxacin | Yes |  |  |  |  |  |  |  |  |  |
| Fluoroquinolones | Watch | Moxifloxacin | Yes |  |  |  |  |  |  |  |  |  |
| Fluoroquinolones | Watch | Norfloxacin | No |  |  |  |  |  |  |  |  |  |
| Macrolides | Watch | Azithromycin | Yes |  |  |  |  |  |  |  |  |  |
| Macrolides | Watch | Clarithromycin | No |  |  |  |  |  |  |  |  |  |
| Macrolides | Watch | Erythromycin | Yes |  |  |  |  |  |  |  |  |  |
| Macrolides | Watch | Roxithromycin | No |  |  |  |  |  |  |  |  |  |
| Tetracyclines | Watch | Minocycline | Yes |  |  |  |  |  |  |  |  |  |
| Rifamycins | Watch | Rifampicin | Yes |  |  |  |  |  |  |  |  |  |
| Rifamycins | Watch | Rifamycin | No |  |  |  |  |  |  |  |  |  |
| Rifamycins | Watch | Rifaximin | No |  |  |  |  |  |  |  |  |  |
| Glycopeptides | Watch | Teicoplanin | No |  |  |  |  |  |  |  |  |  |
| Glycopeptides | Watch | Vancomycin | Yes |  |  |  |  |  |  |  |  |  |
| Third-generation cephalosporins | Reserve | Ceftazidime-avibactam | Yes |  |  |  |  |  |  |  |  |  |
| Fifth-generation cephalosporins | Reserve | Ceftolozane-tazobactam | Yes |  |  |  |  |  |  |  |  |  |
| Carbapenems | Reserve | Meropenem-vaborbactam | Yes |  |  |  |  |  |  |  |  |  |
| Monobactams | Reserve | Aztreonam | Yes |  |  |  |  |  |  |  |  |  |
| Glycylcyclines | Reserve | Tigecycline | Yes |  |  |  |  |  |  |  |  |  |
| Lipopeptides | Reserve | Daptomycin | Yes |  |  |  |  |  |  |  |  |  |
| Oxazolidinones | Reserve | Linezolid | Yes |  |  |  |  |  |  |  |  |  |
| Phosphonics | Reserve | Fosfomycin(IV) | Yes |  |  |  |  |  |  |  |  |  |
| Polymyxins | Reserve | Colistin | Yes |  |  |  |  |  |  |  |  |  |
| Polymyxins | Reserve | Polymyxin B | Yes |  |  |  |  |  |  |  |  |  |
| Antimalarials | NA | Hydroxychloroquine | Yes |  |  |  |  |  |  |  |  |  |

*Cells highlighted in ‘green’ indicate the usage of respective antimicrobials.

**Table S9. Differences in prescription of different antimicrobials between pre-pandemic and pandemic periods in Bangladesh.**

| **Category** | **Name of antimicrobials** | **Pre-pandemic (n=416)** | **Pandemic (n=1780)** | ***p* value^1^** | **OR** | **95% CI** |
| --- | --- | --- | --- | --- | --- | --- |
| Access | Amikacin | 34 (8.2) | 30 (1.7) | <0.0001 | 0.193 | 0.116-0.319 |
|  | Amoxicillin | 3 (0.7) | 2 (0.1) | 0.0190 | 0.155 | 0.026-0.930 |
|  | Amoxicillin/clavulanic Acid | 125 (30) | 641 (36) | 0.0216 | 1.310 | 1.040-1.650 |
|  | Ampicillin | 0 (0) | 1 (0.1) | - | - | - |
|  | Cefradine | 0 (0) | 1 (0.1) | - | - | - |
|  | Clindamycin | 37 (8.9) | 68 (3.8) | <0.0001 | 0.407 | 0.269-0.616 |
|  | Doxycycline | 0 (0) | 16 (0.9) | - | - | - |
|  | Flucloxacillin | 8 (1.9) | 19 (1.1) | 0.1539 | 0.550 | 0.239-1.266 |
|  | Gentamicin | 0 (0) | 3 (0.2) | - | - | - |
|  | Metronidazole | 56 (13.5) | 94 (5.3) | <0.0001 | 0.358 | 0.253-0.509 |
|  | Nitrofurantoin | 0 (0) | 1 (0.1) | - | - | - |
|  | Phenoxymethylpenicillin | 0 (0) | 1 (0.1) | - | - | - |
|  | Sulfamethoxazole/trimethoprim | 1 (0.2) | 3 (0.2) | 0.7570 | 0.701 | 0.073-6.752 |
| Watch | Azithromycin | 7 (1.7) | 180 (10.1) | <0.0001 | 6.573 | 3.065-14.095 |
|  | Cefepime | 0 (0) | 6 (0.3) | - | - | - |
|  | Cefixime | 5 (1.2) | 61 (3.4) | 0.0167 | 2.917 | 1.165-7.305 |
|  | Cefotaxime | 0 (0) | 2 (0.1) | - | - | - |
|  | Ceftazidime | 8 (1.9) | 60 (3.4) | 0.1249 | 1.779 | 0.844-3.750 |
|  | Ceftibuten | 0 (0) | 1 (0.1) | - | - | - |
|  | Ceftriaxone | 266 (63.9) | 727 (40.8) | <0.0001 | 0.389 | 0.312-0.486 |
|  | Cefuroxime | 3 (0.7) | 15 (0.8) | 0.8045 | 1.170 | 0.337-4.060 |
|  | Ciprofloxacin | 5 (1.2) | 29 (1.6) | 0.5251 | 1.361 | 0.524-3.538 |
|  | Clarithromycin | 131 (31.5) | 210 (11.8) | <0.0001 | 0.291 | 0.226-0.374 |
|  | Doripenem | 0 (0) | 2 (0.1) | - | - | - |
|  | Ertapenem | 1 (0.2) | 0 (0) | - | - | - |
|  | Erythromycin | 1 (0.2) | 1 (0.1) | 0.2621 | 0.233 | 0.015-3.737 |
|  | Imipenem/cilastatin | 0 (0) | 1 (0.1) | - | - | - |
|  | Levofloxacin | 19 (4.6) | 45 (2.5) | 0.0260 | 0.542 | 0.314-0.937 |
|  | Meropenem | 65 (15.6) | 497 (27.9) | <0.0001 | 2.092 | 1.574-2.779 |
|  | Moxifloxacin | 15 (3.6) | 492 (27.6) | <0.0001 | 10.212 | 6.036-17.276 |
|  | Piperacillin | 0 (0) | 1 (0.1) | - | - | - |
|  | Piperacillin/tazobactam | 4 (1) | 37 (2.1) | 0.1296 | 2.186 | 0.775-6.168 |
|  | Rifaximin | 5 (1.2) | 5 (0.3) | 0.0120 | 0.232 | 0.067-0.804 |
|  | Teicoplanin | 0 (0) | 8 (0.4) | - | - | - |
|  | Vancomycin | 11 (2.6) | 12 (0.7) | <0.0001 | 0.250 | 0.109-0.570 |
| Reserve | Colistin | 2 (0.5) | 26 (1.5) | 0.1088 | 3.068 | 0.725-12.979 |
|  | Linezolid | 4 (1) | 76 (4.3) | 0.0012 | 4.594 | 1.671-12.628 |
|  | Polymyxin B | 0 (0) | 10 (0.6) | - | - | - |
|  | Tigecycline | 2 (0.5) | 9 (0.5) | 0.9485 | 1.052 | 0.226-4.887 |

^1^Obtained from an unadjusted Chi-squared/Fisher’s exact test.

**Table S10. Differences in prescription of different antimicrobials between pre-pandemic and pandemic periods in Brazil.**

| **Category** | **Name of antimicrobials** | **Pre-pandemic (n=161)** | **Pandemic (n=1131)** | ***p* value^1^** | **OR** | **95% CI** |
| --- | --- | --- | --- | --- | --- | --- |
| Access | Amikacin | 10 (6.2) | 202 (17.9) | <0.0001 | 3.283 | 1.701-6.339 |
|  | Amoxicillin | 1 (0.6) | 1 (0.1) | 0.1077 | 0.142 | 0.009-2.275 |
|  | Amoxicillin/clavulanic Acid | 1 (0.6) | 2 (0.2) | 0.2731 | 0.283 | 0.026-3.144 |
|  | Ampicillin | 2 (1.2) | 4 (0.4) | 0.1208 | 0.282 | 0.051-1.553 |
|  | Ampicillin/sulbactam | 1 (0.6) | 12 (1.1) | 0.6008 | 1.716 | 0.222-13.285 |
|  | Cefalotin | 0 (0) | 1 (0.1) | - | - | - |
|  | Cefazolin | 3 (1.9) | 4 (0.4) | 0.0146 | 0.187 | 0.041-0.843 |
|  | Cefroxadine | 0 (0) | 1 (0.1) | - | - | - |
|  | Clindamycin | 7 (4.3) | 24 (2.1) | 0.0842 | 0.477 | 0.202-1.126 |
|  | Gentamicin | 2 (1.2) | 8 (0.7) | 0.4687 | 0.566 | 0.119-2.691 |
|  | Metronidazole | 6 (3.7) | 32 (2.8) | 0.5284 | 0.752 | 0.310-1.828 |
|  | Oxacillin | 8 (5) | 15 (1.3) | 0.0011 | 0.257 | 0.107-0.616 |
|  | Sulfadiazine/trimethoprim | 2 (1.2) | 3 (0.3) | 0.0618 | 0.211 | 0.035-1.275 |
|  | Sulfamethizole/trimethoprim | 1 (0.6) | 3 (0.3) | 0.4470 | 0.426 | 0.044-4.116 |
|  | Sulfamethoxazole/trimethoprim | 3 (1.9) | 74 (6.5) | 0.0189 | 3.687 | 1.149-11.837 |
|  | Sulfametrole/trimethoprim | 1 (0.6) | 1 (0.1) | 0.1077 | 0.142 | 0.009-2.275 |
|  | Sulfamoxole/trimethoprim | 0 (0) | 4 (0.4) | - | - | - |
| Watch | Azithromycin | 11 (6.8) | 508 (44.9) | <0.0001 | 11.119 | 5.962-20.739 |
|  | Cefepime | 3 (1.9) | 36 (3.2) | 0.3598 | 1.732 | 0.527-5.689 |
|  | Cefotaxime | 0 (0) | 1 (0.1) | - | - | - |
|  | Ceftazidime | 4 (2.5) | 16 (1.4) | 0.3036 | 0.563 | 0.186-1.706 |
|  | Ceftriaxone | 59 (36.6) | 716 (63.3) | <0.0001 | 2.983 | 2.117-4.202 |
|  | Ciprofloxacin | 1 (0.6) | 16 (1.4) | 0.4084 | 2.296 | 0.302-17.430 |
|  | Clarithromycin | 30 (18.6) | 176 (15.6) | 0.3191 | 0.805 | 0.525-1.235 |
|  | Ertapenem | 0 (0) | 14 (1.2) | - | - | - |
|  | Levofloxacin | 5 (3.1) | 64 (5.7) | 0.1776 | 1.871 | 0.742-4.722 |
|  | Meropenem | 68 (42.2) | 502 (44.4) | 0.6073 | 1.092 | 0.782-1.524 |
|  | Norfloxacin | 0 (0) | 1 (0.1) | - | - | - |
|  | Piperacillin | 0 (0) | 9 (0.8) | - | - | - |
|  | Piperacillin/tazobactam | 55 (34.2) | 273 (24.1) | 0.0063 | 0.613 | 0.431-0.873 |
|  | Teicoplanin | 0 (0) | 2 (0.2) | - | - | - |
|  | Vancomycin | 69 (42.9) | 437 (38.6) | 0.3049 | 0.840 | 0.601-1.173 |
| Reserve | Ceftazidime-avibactam | 1 (0.6) | 1 (0.1) | 0.1077 | 0.142 | 0.009-2.275 |
|  | Fosfomycin | 0 (0) | 1 (0.1) | - | - | - |
|  | Linezolid | 2 (1.2) | 37 (3.3) | 0.1591 | 2.689 | 0.642-11.264 |
|  | Polymyxin B | 22 (13.7) | 264 (23.3) | 0.0057 | 1.924 | 1.202-3.079 |
|  | Tigecycline | 1 (0.6) | 31 (2.7) | 0.1054 | 4.509 | 0.611-33.259 |

^1^Obtained from an unadjusted Chi-squared/Fisher’s exact test.

**Table S11. Differences in prescription of different antimicrobials between pre-pandemic and pandemic periods in India.**

| **Category** | **Name of antimicrobials** | **Pre-pandemic (n=760)** | **Pandemic (n=1837)** | ***p* value^1^** | **OR** | **95% CI** |
| --- | --- | --- | --- | --- | --- | --- |
| Access | Amikacin | 19 (2.5) | 47 (2.6) | 0.9313 | 1.024 | 0.597-1.757 |
|  | Amoxicillin | 0 (0) | 3 (0.2) | - | - | - |
|  | Amoxicillin/clavulanic Acid | 19 (2.5) | 40 (2.2) | 0.6157 | 0.868 | 0.499-1.509 |
|  | Ampicillin | 12 (1.6) | 24 (1.3) | 0.5890 | 0.825 | 0.411-1.659 |
|  | Ampicillin/sulbactam | 0 (0) | 9 (0.5) | - | - | - |
|  | Benzylpenicillin | 1 (0.1) | 0 (0) | - | - | - |
|  | Clindamycin | 19 (2.5) | 26 (1.4) | 0.0539 | 0.560 | 0.308-1.018 |
|  | Cloxacillin | 5 (0.7) | 1 (0.1) | 0.0036 | 0.082 | 0.010-0.705 |
|  | Dicloxacillin | 14 (1.8) | 22 (1.2) | 0.2012 | 0.646 | 0.329-1.269 |
|  | Doxycycline | 21 (2.8) | 53 (2.9) | 0.8650 | 1.045 | 0.626-1.746 |
|  | Gentamicin | 9 (1.2) | 3 (0.2) | 0.0004 | 0.136 | 0.037-0.506 |
|  | Metronidazole | 4 (0.5) | 5 (0.3) | 0.3160 | 0.516 | 0.138-1.926 |
|  | Sulfamethoxazole/trimethoprim | 3 (0.4) | 10 (0.5) | 0.6230 | 1.381 | 0.379-5.032 |
| Watch | Azithromycin | 41 (5.4) | 92 (5) | 0.6843 | 0.925 | 0.633-1.349 |
|  | Cefbuperazone | 7 (0.9) | 17 (0.9) | 0.9916 | 1.005 | 0.415-2.433 |
|  | Cefodizime | 0 (0) | 1 (0.1) | - | - | - |
|  | Cefoperazone | 43 (5.7) | 66 (3.6) | 0.0170 | 0.621 | 0.419-0.921 |
|  | Cefotaxime | 15 (2) | 17 (0.9) | 0.0280 | 0.464 | 0.230-0.934 |
|  | Ceftazidime | 17 (2.2) | 40 (2.2) | 0.9251 | 0.973 | 0.548-1.727 |
|  | Ceftriaxone | 13 (1.7) | 27 (1.5) | 0.6504 | 0.857 | 0.440-1.670 |
|  | Cefuroxime | 9 (1.2) | 22 (1.2) | 0.9772 | 1.011 | 0.464-2.207 |
|  | Ciprofloxacin | 7 (0.9) | 13 (0.7) | 0.5714 | 0.767 | 0.305-1.929 |
|  | Ertapenem | 7 (0.9) | 9 (0.5) | 0.2014 | 0.530 | 0.197-1.427 |
|  | Imipenem/cilastatin | 2 (0.3) | 2 (0.1) | 0.3617 | 0.413 | 0.058-2.938 |
|  | Levofloxacin | 59 (7.8) | 112 (6.1) | 0.1193 | 0.771 | 0.556-1.070 |
|  | Meropenem | 568 (74.7) | 1470 (80) | 0.0029 | 1.354 | 1.109-1.653 |
|  | Norfloxacin | 2 (0.3) | 0 (0) | - | - | - |
|  | Piperacillin | 1 (0.1) | 6 (0.3) | 0.3831 | 2.487 | 0.299-20.694 |
|  | Piperacillin/tazobactam | 190 (25) | 462 (25.1) | 0.9362 | 1.008 | 0.829-1.225 |
|  | Rifampicin | 1 (0.1) | 0 (0) | - | - | - |
|  | Sulbenicillin | 0 (0) | 1 (0.1) | - | - | - |
|  | Teicoplanin | 40 (5.3) | 154 (8.4) | 0.0059 | 1.647 | 1.151-2.357 |
|  | Vancomycin | 59 (7.8) | 151 (8.2) | 0.6977 | 1.064 | 0.778-1.456 |
| Reserve | Aztreonam | 3 (0.4) | 9 (0.5) | 0.7448 | 1.242 | 0.335-4.602 |
|  | Ceftazidime-avibactam | 3 (0.4) | 3 (0.2) | 0.2637 | 0.413 | 0.083-2.050 |
|  | Colistin | 2 (0.3) | 0 (0) | - | - | - |
|  | Fosfomycin | 1 (0.1) | 0 (0) | - | - | - |
|  | Linezolid | 4 (0.5) | 17 (0.9) | 0.3015 | 1.765 | 0.592-5.264 |
|  | Meropenem-vaborbactam | 0 (0) | 3 (0.2) | - | - | - |
|  | Tigecycline | 26 (3.4) | 100 (5.4) | 0.0291 | 1.625 | 1.047-2.524 |

^1^Obtained from an unadjusted Chi-squared/Fisher’s exact test.

**Table S12. Differences in prescription of different antimicrobials between pre-pandemic and pandemic periods in Italy.**

| **Category** | **Name of antimicrobials** | **Pre-pandemic (n=490)** | **Pandemic (n=1902)** | ***p* value^1^** | **OR** | **95% CI** |
| --- | --- | --- | --- | --- | --- | --- |
| Access | Amikacin | 19 (3.9) | 59 (3.1) | 0.3887 | 0.794 | 0.469-1.344 |
|  | Amoxicillin | 14 (2.9) | 8 (0.4) | <0.0001 | 0.144 | 0.060-0.344 |
|  | Amoxicillin/clavulanic Acid | 48 (9.8) | 136 (7.2) | 0.0500 | 0.709 | 0.502-1.001 |
|  | Ampicillin | 17 (3.5) | 62 (3.3) | 0.8169 | 0.938 | 0.543-1.619 |
|  | Ampicillin/sulbactam | 0 (0) | 1 (0.1) | - | - | - |
|  | Cefazolin | 14 (2.9) | 24 (1.3) | 0.0118 | 0.435 | 0.223-0.846 |
|  | Clindamycin | 7 (1.4) | 20 (1.1) | 0.4811 | 0.733 | 0.308-1.744 |
|  | Doxycycline | 0 (0) | 4 (0.2) | - | - | - |
|  | Gentamicin | 11 (2.2) | 16 (0.8) | 0.0087 | 0.369 | 0.170-0.801 |
|  | Metronidazole | 15 (3.1) | 42 (2.2) | 0.2696 | 0.715 | 0.393-1.300 |
|  | Oxacillin | 35 (7.1) | 68 (3.6) | 0.0005 | 0.482 | 0.317-0.734 |
|  | Sulfamethizole/trimethoprim | 1 (0.2) | 0 (0) | - | - | - |
|  | Sulfamethoxazole/trimethoprim | 7 (1.4) | 52 (2.7) | 0.0967 | 1.939 | 0.875-4.297 |
|  | Sulfametrole/trimethoprim | 0 (0) | 2 (0.1) | - | - | - |
| Watch | Azithromycin | 39 (8) | 152 (8) | 0.9812 | 1.004 | 0.696-1.449 |
|  | Cefepime | 6 (1.2) | 19 (1) | 0.6616 | 0.814 | 0.323-2.049 |
|  | Cefixime | 6 (1.2) | 10 (0.5) | 0.0906 | 0.426 | 0.154-1.179 |
|  | Cefoxitin | 0 (0) | 2 (0.1) | - | - | - |
|  | Ceftazidime | 5 (1) | 23 (1.2) | 0.7289 | 1.187 | 0.449-3.139 |
|  | Ceftriaxone | 210 (42.9) | 830 (43.6) | 0.7558 | 1.032 | 0.845-1.262 |
|  | Ciprofloxacin | 10 (2) | 23 (1.2) | 0.1594 | 0.588 | 0.278-1.243 |
|  | Clarithromycin | 2 (0.4) | 5 (0.3) | 0.5955 | 0.643 | 0.124-3.325 |
|  | Ertapenem | 1 (0.2) | 1 (0.1) | 0.3008 | 0.257 | 0.016-4.120 |
|  | Imipenem/cilastatin | 1 (0.2) | 4 (0.2) | 0.9785 | 1.031 | 0.115-9.241 |
|  | Levofloxacin | 41 (8.4) | 134 (7) | 0.3162 | 0.830 | 0.576-1.195 |
|  | Meropenem | 55 (11.2) | 203 (10.7) | 0.7256 | 0.945 | 0.689-1.296 |
|  | Piperacillin | 3 (0.6) | 1 (0.1) | 0.0069 | 0.085 | 0.009-0.823 |
|  | Piperacillin/tazobactam | 202 (41.2) | 868 (45.6) | 0.0799 | 1.197 | 0.979-1.464 |
|  | Rifampicin | 8 (1.6) | 4 (0.2) | <0.0001 | 0.127 | 0.038-0.423 |
|  | Rifamycin | 0 (0) | 1 (0.1) | - | - | - |
|  | Rifaximin | 1 (0.2) | 2 (0.1) | 0.5811 | 0.515 | 0.047-5.688 |
|  | Teicoplanin | 7 (1.4) | 29 (1.5) | 0.8761 | 1.068 | 0.465-2.454 |
|  | Tobramycin | 3 (0.6) | 1 (0.1) | 0.0069 | 0.085 | 0.009-0.823 |
|  | Vancomycin | 45 (9.2) | 186 (9.8) | 0.6907 | 1.072 | 0.761-1.509 |
| Reserve | Ceftazidime-avibactam | 0 (0) | 4 (0.2) | - | - | - |
|  | Ceftolozane-tazobactam | 0 (0) | 1 (0.1) | - | - | - |
|  | Daptomycin | 5 (1) | 28 (1.5) | 0.4446 | 1.449 | 0.557-3.773 |
|  | Fosfomycin | 4 (0.8) | 16 (0.8) | 0.9570 | 1.031 | 0.343-3.097 |
|  | Linezolid | 3 (0.6) | 34 (1.8) | 0.0601 | 2.955 | 0.904-9.661 |
|  | Tigecycline | 0 (0) | 2 (0.1) | - | - | - |
| NA^2^ | Hydroxychloroquine | 0 (0) | 35 (1.8) | - | - | - |

^1^Obtained from an unadjusted Chi-squared/Fisher’s exact test. ^2^Antimicrobials that are not assigned in AWaRe groups.

**Table S13. Differences in prescription of different antimicrobials between pre-pandemic and pandemic periods in Nigeria.**

| **Category** | **Name of antimicrobials** | **Pre-pandemic (n=17)** | **Pandemic (n=468)** | ***p* value^1^** | **OR** | **95% CI** |
| --- | --- | --- | --- | --- | --- | --- |
| Access | Amoxicillin/clavulanic Acid | 9 (52.9) | 293 (62.6) | 0.4193 | 1.488 | 0.564-3.928 |
|  | Ampicillin | 0 (0) | 1 (0.2) | - | - | - |
|  | Metronidazole | 0 (0) | 20 (4.3) | - | - | - |
|  | Sulfamethoxazole/trimethoprim | 1 (5.9) | 5 (1.1) | 0.0777 | 0.173 | 0.019-1.566 |
| Watch | Azithromycin | 12 (70.6) | 392 (83.8) | 0.1526 | 2.149 | 0.736-6.277 |
|  | Azlocillin | 0 (0) | 2 (0.4) | - | - | - |
|  | Cefixime | 1 (5.9) | 1 (0.2) | 0.0003 | 0.034 | 0.002-0.573 |
|  | Cefpodoxime proxetil | 0 (0) | 1 (0.2) | - | - | - |
|  | Ceftazidime | 0 (0) | 1 (0.2) | - | - | - |
|  | Ceftriaxone | 12 (70.6) | 181 (38.7) | 0.0083 | 0.263 | 0.091-0.758 |
|  | Cefuroxime | 1 (5.9) | 5 (1.1) | 0.0777 | 0.173 | 0.019-1.566 |
|  | Ciprofloxacin | 2 (11.8) | 8 (1.7) | 0.0042 | 0.130 | 0.025-0.667 |
|  | Imipenem/cilastatin | 0 (0) | 1 (0.2) | - | - | - |
|  | Levofloxacin | 2 (11.8) | 2 (0.4) | <0.0001 | 0.032 | 0.004-0.244 |
|  | Meropenem | 0 (0) | 20 (4.3) | - | - | - |
|  | Moxifloxacin | 0 (0) | 3 (0.6) | - | - | - |
|  | Piperacillin/tazobactam | 1 (5.9) | 0 (0) | - | - | - |
|  | Rifampicin | 0 (0) | 1 (0.2) | - | - | - |
|  | Vancomycin | 0 (0) | 4 (0.9) | - | - | - |
| NA | Hydroxychloroquine | 0 (0) | 2 (0.4) | - | - | - |

^1^Obtained from an unadjusted Chi-squared/Fisher’s exact test. ^2^Antimicrobials that are not assigned in AWaRe groups.

**Table S14. Differences in prescription of different antimicrobials between pre-pandemic and pandemic periods in South Korea.**

| **Category** | **Name of antimicrobials** | **Pre-pandemic (n=246)** | **Pandemic (n=1789)** | ***p* value^1^** | **OR** | **95% CI** |
| --- | --- | --- | --- | --- | --- | --- |
| Access | Amoxicillin | 1 (0.4) | 29 (1.6) | 0.1383 | 4.037 | 0.547-29.768 |
|  | Ampicillin | 5 (2) | 24 (1.3) | 0.3912 | 0.655 | 0.248-1.734 |
|  | Clindamycin | 3 (1.2) | 27 (1.5) | 0.7237 | 1.241 | 0.374-4.122 |
|  | Doxycycline | 1 (0.4) | 8 (0.4) | 0.9282 | 1.101 | 0.137-8.837 |
|  | Metronidazole | 12 (4.9) | 164 (9.2) | 0.0248 | 1.968 | 1.078-3.593 |
|  | Sulfamethizole/trimethoprim | 0 (0) | 3 (0.2) | - | - | - |
|  | Sulfamethoxazole/trimethoprim | 51 (20.7) | 536 (30) | 0.0027 | 1.636 | 1.182-2.262 |
| Watch | Arbekacin | 0 (0) | 2 (0.1) | - | - | - |
|  | Azithromycin | 9 (3.7) | 42 (2.3) | 0.2175 | 0.633 | 0.304-1.317 |
|  | Cefaclor | 0 (0) | 2 (0.1) | - | - | - |
|  | Cefepime | 26 (10.6) | 305 (17) | 0.0098 | 1.739 | 1.137-2.659 |
|  | Cefixime | 0 (0) | 1 (0.1) | - | - | - |
|  | Cefoperazone | 7 (2.8) | 34 (1.9) | 0.3226 | 0.661 | 0.290-1.509 |
|  | Cefotetan | 2 (0.8) | 22 (1.2) | 0.5703 | 1.519 | 0.355-6.500 |
|  | Cefotiam | 8 (3.3) | 20 (1.1) | 0.0071 | 0.336 | 0.147-0.772 |
|  | Cefpiramide | 2 (0.8) | 7 (0.4) | 0.3500 | 0.479 | 0.099-2.320 |
|  | Cefpodoxime proxetil | 2 (0.8) | 13 (0.7) | 0.8820 | 0.893 | 0.200-3.981 |
|  | Cefprozil | 0 (0) | 2 (0.1) | - | - | - |
|  | Ceftazidime | 12 (4.9) | 59 (3.3) | 0.2054 | 0.665 | 0.352-1.256 |
|  | Ceftriaxone | 36 (14.6) | 293 (16.4) | 0.4861 | 1.142 | 0.785-1.662 |
|  | Ciprofloxacin | 16 (6.5) | 99 (5.5) | 0.5366 | 0.842 | 0.488-1.453 |
|  | Clarithromycin | 1 (0.4) | 5 (0.3) | 0.7305 | 0.687 | 0.080-5.902 |
|  | Ertapenem | 1 (0.4) | 13 (0.7) | 0.5689 | 1.793 | 0.234-13.769 |
|  | Flomoxef | 11 (4.5) | 73 (4.1) | 0.7725 | 0.909 | 0.475-1.738 |
|  | Isepamicin | 2 (0.8) | 0 (0) | - | - | - |
|  | Levofloxacin | 102 (41.5) | 751 (42) | 0.8779 | 1.021 | 0.779-1.339 |
|  | Meropenem | 100 (40.7) | 695 (38.8) | 0.5871 | 0.928 | 0.707-1.217 |
|  | Minocycline | 8 (3.3) | 68 (3.8) | 0.6703 | 1.175 | 0.558-2.476 |
|  | Moxifloxacin | 5 (2) | 39 (2.2) | 0.8815 | 1.074 | 0.419-2.752 |
|  | Netilmicin | 0 (0) | 2 (0.1) | - | - | - |
|  | Piperacillin | 0 (0) | 1 (0.1) | - | - | - |
|  | Piperacillin/tazobactam | 79 (32.1) | 699 (39.1) | 0.0352 | 1.356 | 1.020-1.801 |
|  | Rifampicin | 4 (1.6) | 23 (1.3) | 0.6618 | 0.788 | 0.270-2.298 |
|  | Rifaximin | 6 (2.4) | 65 (3.6) | 0.3385 | 1.508 | 0.646-3.518 |
|  | Roxithromycin | 0 (0) | 1 (0.1) | - | - | - |
|  | Teicoplanin | 134 (54.5) | 912 (51) | 0.3040 | 0.869 | 0.665-1.136 |
|  | Tobramycin | 1 (0.4) | 4 (0.2) | 0.5869 | 0.549 | 0.061-4.932 |
|  | Vancomycin | 37 (15) | 235 (13.1) | 0.4104 | 0.854 | 0.587-1.244 |
|  | Gemifloxacin | 0 (0) | 1 (0.1) | - | - | - |
| Reserve | Aztreonam | 3 (1.2) | 27 (1.5) | 0.7237 | 1.241 | 0.374-4.122 |
|  | Colistin | 10 (4.1) | 103 (5.8) | 0.2771 | 1.442 | 0.743-2.799 |
|  | Daptomycin | 0 (0) | 2 (0.1) | - | - | - |
|  | Linezolid | 10 (4.1) | 57 (3.2) | 0.4689 | 0.777 | 0.391-1.542 |
|  | Tigecycline | 2 (0.8) | 22 (1.2) | 0.5703 | 1.519 | 0.355-6.500 |

^1^Obtained from an unadjusted Chi-squared/Fisher’s exact test.

**Table S15. Differences in prescription of different antimicrobials between pre-pandemic and pandemic periods in Switzerland.**

| **Category** | **Name of antimicrobials** | **Pre-pandemic (n=259)** | **Pandemic (n=932)** | ***p* value^1^** | **OR** | **95% CI** |
| --- | --- | --- | --- | --- | --- | --- |
| Access | Amikacin | 6 (2.3) | 31 (3.3) | 0.4070 | 1.451 | 0.599-3.516 |
|  | Amoxicillin | 7 (2.7) | 26 (2.8) | 0.9400 | 1.033 | 0.443-2.408 |
|  | Amoxicillin/clavulanic Acid | 88 (34) | 594 (63.7) | <0.0001 | 3.415 | 2.556-4.563 |
|  | Ampicillin | 2 (0.8) | 0 (0) | - | - | - |
|  | Benzylpenicillin | 5 (1.9) | 3 (0.3) | 0.0050 | 0.164 | 0.039-0.691 |
|  | Cefazolin | 2 (0.8) | 14 (1.5) | 0.3670 | 1.960 | 0.443-8.678 |
|  | Clindamycin | 8 (3.1) | 13 (1.4) | 0.0670 | 0.444 | 0.182-1.083 |
|  | Doxycycline | 0 (0) | 5 (0.5) | - | - | - |
|  | Flucloxacillin | 2 (0.8) | 10 (1.1) | 0.6680 | 1.394 | 0.303-6.401 |
|  | Gentamicin | 2 (0.8) | 10 (1.1) | 0.6680 | 1.394 | 0.303-6.401 |
|  | Metronidazole | 31 (12) | 33 (3.5) | <0.0001 | 0.270 | 0.162-0.450 |
|  | Nitrofurantoin | 0 (0) | 14 (1.5) | - | - | - |
|  | Phenoxymethylpenicillin | 0 (0) | 1 (0.1) | - | - | - |
|  | Sulfamethizole/trimethoprim | 1 (0.4) | 0 (0) | - | - | - |
|  | Sulfamethoxazole/trimethoprim | 7 (2.7) | 19 (2) | 0.5180 | 0.749 | 0.311-1.802 |
| Watch | Azithromycin | 0 (0) | 46 (4.9) | - | - | - |
|  | Cefepime | 11 (4.2) | 47 (5) | 0.5990 | 1.197 | 0.612-2.343 |
|  | Cefoperazone | 0 (0) | 1 (0.1) | - | - | - |
|  | Ceftazidime | 0 (0) | 9 (1) | - | - | - |
|  | Ceftibuten | 1 (0.4) | 0 (0) | - | - | - |
|  | Ceftriaxone | 47 (18.1) | 118 (12.7) | 0.0240 | 0.654 | 0.452-0.947 |
|  | Cefuroxime | 8 (3.1) | 26 (2.8) | 0.7980 | 0.900 | 0.403-2.013 |
|  | Ciprofloxacin | 7 (2.7) | 43 (4.6) | 0.1750 | 1.741 | 0.774-3.918 |
|  | Clarithromycin | 55 (21.2) | 117 (12.6) | 0.0004 | 0.532 | 0.373-0.760 |
|  | Ertapenem | 0 (0) | 2 (0.2) | - | - | - |
|  | Erythromycin | 0 (0) | 5 (0.5) | - | - | - |
|  | Imipenem/cilastatin | 36 (13.9) | 79 (8.5) | 0.0090 | 0.574 | 0.377-0.874 |
|  | Levofloxacin | 11 (4.2) | 60 (6.4) | 0.1880 | 1.551 | 0.803-2.996 |
|  | Meropenem | 12 (4.6) | 37 (4) | 0.6350 | 0.851 | 0.437-1.657 |
|  | Moxifloxacin | 0 (0) | 2 (0.2) | - | - | - |
|  | Piperacillin | 0 (0) | 34 (3.6) | - | - | - |
|  | Piperacillin/tazobactam | 110 (42.5) | 292 (31.3) | 0.0008 | 0.618 | 0.466-0.820 |
|  | Rifampicin | 0 (0) | 2 (0.2) | - | - | - |
|  | Tobramycin | 0 (0) | 2 (0.2) | - | - | - |
|  | Vancomycin | 39 (15.1) | 86 (9.2) | 0.0070 | 0.573 | 0.382-0.861 |
| Reserve | Aztreonam | 0 (0) | 7 (0.8) | - | - | - |
|  | Ceftazidime-avibactam | 0 (0) | 2 (0.2) | - | - | - |
|  | Colistin | 0 (0) | 2 (0.2) | - | - | - |
|  | Daptomycin | 1 (0.4) | 6 (0.6) | 0.6310 | 1.672 | 0.200-13.948 |
|  | Fosfomycin | 0 (0) | 2 (0.2) | - | - | - |
|  | Linezolid | 0 (0) | 2 (0.2) | - | - | - |
| NA | Hydroxychloroquine | 0 (0) | 83 (8.9) | - | - | - |

^1^Obtained from an unadjusted Chi-squared/Fisher’s exact test. ^2^Antimicrobials that are not assigned in AWaRe groups.

**Table S16. Differences in prescription of different antimicrobials between pre-pandemic and pandemic periods in Turkey.**

| **Category** | **Name of antimicrobials** | **Pre-pandemic (n=279)** | **Pandemic (n=1176)** | ***p* value^1^** | **OR** | **95% CI** |
| --- | --- | --- | --- | --- | --- | --- |
| Access | Amikacin | 8 (2.9) | 79 (6.7) | 0.0147 | 2.439 | 1.165-5.109 |
|  | Amoxicillin/clavulanic Acid | 3 (1.1) | 4 (0.3) | 0.1106 | 0.314 | 0.070-1.411 |
|  | Ampicillin | 0 (0) | 9 (0.8) | - | - | - |
|  | Ampicillin/sulbactam | 12 (4.3) | 43 (3.7) | 0.6118 | 0.844 | 0.439-1.623 |
|  | Cefazolin | 21 (7.5) | 53 (4.5) | 0.0390 | 0.580 | 0.344-0.978 |
|  | Clindamycin | 7 (2.5) | 12 (1) | 0.0490 | 0.401 | 0.156-1.027 |
|  | Gentamicin | 0 (0) | 8 (0.7) | - | - | - |
|  | Metronidazole | 11 (3.9) | 26 (2.2) | 0.0986 | 0.551 | 0.269-1.129 |
|  | Sulfamethoxazole/trimethoprim | 10 (3.6) | 41 (3.5) | 0.9363 | 0.972 | 0.481-1.964 |
| Watch | Azithromycin | 0 (0) | 11 (0.9) | - | - | - |
|  | Cefepime | 9 (3.2) | 39 (3.3) | 0.9393 | 1.029 | 0.493-2.150 |
|  | Cefixime | 0 (0) | 5 (0.4) | - | - | - |
|  | Cefoperazone | 8 (2.9) | 9 (0.8) | 0.0033 | 0.261 | 0.100-0.683 |
|  | Cefotaxime | 2 (0.7) | 3 (0.3) | 0.2361 | 0.354 | 0.059-2.130 |
|  | Ceftazidime | 16 (5.7) | 20 (1.7) | 0.0001 | 0.284 | 0.145-0.556 |
|  | Ceftriaxone | 34 (12.2) | 146 (12.4) | 0.9170 | 1.021 | 0.686-1.521 |
|  | Cefuroxime | 5 (1.8) | 5 (0.4) | 0.0130 | 0.234 | 0.067-0.814 |
|  | Ciprofloxacin | 12 (4.3) | 28 (2.4) | 0.0778 | 0.543 | 0.272-1.081 |
|  | Clarithromycin | 11 (3.9) | 30 (2.6) | 0.2067 | 0.638 | 0.316-1.289 |
|  | Ertapenem | 21 (7.5) | 86 (7.3) | 0.9020 | 0.969 | 0.590-1.592 |
|  | Imipenem/cilastatin | 14 (5) | 45 (3.8) | 0.3644 | 0.753 | 0.407-1.392 |
|  | Levofloxacin | 17 (6.1) | 91 (7.7) | 0.3460 | 1.293 | 0.757-2.207 |
|  | Meropenem | 157 (56.3) | 757 (64.4) | 0.0119 | 1.404 | 1.077-1.830 |
|  | Moxifloxacin | 10 (3.6) | 12 (1) | 0.0016 | 0.277 | 0.119-0.649 |
|  | Piperacillin | 0 (0) | 1 (0.1) | - | - | - |
|  | Piperacillin/tazobactam | 106 (38) | 506 (43) | 0.1257 | 1.233 | 0.943-1.611 |
|  | Rifampicin | 0 (0) | 1 (0.1) | - | - | - |
|  | Teicoplanin | 65 (23.3) | 276 (23.5) | 0.9514 | 1.010 | 0.742-1.375 |
|  | Vancomycin | 31 (11.1) | 99 (8.4) | 0.1563 | 0.735 | 0.480-1.126 |
| Reserve | Ceftazidime-avibactam | 0 (0) | 7 (0.6) | - | - | - |
|  | Colistin | 70 (25.1) | 213 (18.1) | 0.0081 | 0.660 | 0.485-0.899 |
|  | Daptomycin | 9 (3.2) | 36 (3.1) | 0.8865 | 0.947 | 0.451-1.990 |
|  | Fosfomycin | 26 (9.3) | 67 (5.7) | 0.0262 | 0.588 | 0.366-0.943 |
|  | Linezolid | 23 (8.2) | 90 (7.7) | 0.7403 | 0.922 | 0.572-1.487 |
|  | Polymyxin B | 0 (0) | 23 (2) | - | - | - |
|  | Tigecycline | 31 (11.1) | 124 (10.5) | 0.7826 | 0.943 | 0.621-1.431 |
| NA | Hydroxychloroquine | 0 (0) | 12 (1) | - | - | - |

^1^Obtained from an unadjusted Chi-squared/Fisher’s exact test. ^2^Antimicrobials that are not assigned in AWaRe groups.

**Table S17. Relevant dates to plot antimicrobial usage data.**

| **Country** | **Emergence of Delta variants** | **WHO guideline version 1 released** | **WHO guideline version 2 released** |
| --- | --- | --- | --- |
| Bangladesh | 05/07/2021 | 27/05/2020 | 25/01/2021 |
| Brazil | 04/01/2021 |  |  |
| India | 09/11/2020 |  |  |
| Italy | 04/01/2021 |  |  |
| Malawi | 19/07/2021 |  |  |
| Nigeria | 02/08/2021 |  |  |
| South Korea | 26/04/2021 |  |  |
| Switzerland | 12/04/2021 |  |  |
| Turkey | 15/02/2021 |  |  |

 **Figure S1.** Date of the first reported case of COVID-19 in the countries included in this study ([https://data·who·int/dashboards/covid19/data](https://data.who.int/dashboards/covid19/data)). Each colour box from 20/01/2020 to 02/04/2020 indicates one week and two days.


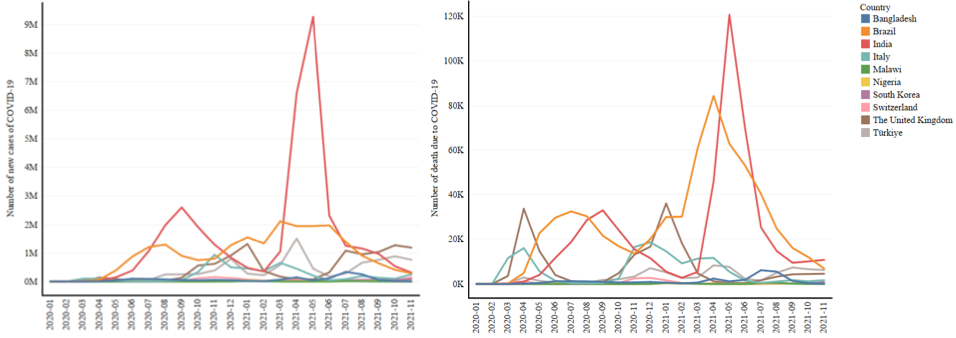


**Figure S2.** a) Number of COVID-19 infected cases and b) number of deaths due to COVID-19 in the countries included in this study during the study period (https://data·who·int/dashboards/covid19/data).

**Figure S3.** The distribution of patients with different age groups among the study population (n=14,002). The number of missing data on age is 56.

**Figure S4.** The distribution of patients with different sexes among the study population (n=14,055). The number of missing data on sex is 3.

**Figure S5.** The distribution of patients with pneumonia and/or sepsis and/or ARDS from studied countries among the study population (n=14,058). Chi-squared tests (unadjusted) were performed to compare the inclusion of patients with different clinical presentations (binary for yes/no against sepsis only/pneumonia only/ARDS only/sepsis and pneumonia/sepsis and ARDS/pneumonia and ARDS/sepsis, pneumonia, and ARDS) among countries (yes/no against the country under investigation vs combined data from other countries). Significantly more cases with ‘pneumonia only’ were included from Bangladesh [89% (1955/2196)] compared to other countries [32% (3791/11862)] (p<0·0001, OR: 0·058, 95% CI: 0·050-0·067), cases with ‘sepsis and ARDS’ from Brazil [13·9% (180/1292)] compared to other countries [0·9% (116/12766)] (p<0·0001, OR: 0·057, 95% CI: 0·045-0·072), cases with ‘sepsis, pneumonia and ARDS’ from Brazil [14·7% (190/1292)] compared to other countries [1·1% (140/12766)] (p<0·0001, OR: 0·064, 95% CI: 0·051-0·081), cases with ‘pneumonia and ARDS’ from India [17·9% (466/2597)] compared to other countries [7·3% (840/11461] (p<0·0001, OR: 0·362, 95% CI: 0·320-0·409), cases with ‘sepsis only’ from Italy [61·4 (1469/ 2392)] compared to other countries [26·5 (3086/11666] (p<0·0001, OR: 0·226, 95% CI: 0·206-0·248).

**Figure S6.** Plot represents the comparison of days of therapy (DOT) of respective antimicrobials between pre-pandemic and pandemic periods at the country level. Horizontal bars represent the lower and upper values of a 95% confidence interval. Black square symbols represent the odds ratio, and red square symbols represent tests with significant differences in the prescription of respective antimicrobials between pre-pandemic and pandemic periods. Antimicrobials with <15 prescriptions overall are excluded from the analysis. Statistical analysis was performed using linear regression. All models adjusted for age (continuous), sex (male/female/other), admitting ward (ICU/HDU/DCC/COVID-specialised [including regular wards for the pre-pandemic period]), comorbidities (binary, yes or no), patient outcome (died/discharged alive) and diagnosis type (sepsis only/pneumonia only/ARDs only/sepsis and pneumonia/sepsis and ARDs/pneumonia and ARDs/sepsis, pneumonia and ARDS).

**Figure S7.** Plot represents the comparison of prescribed daily dose (PDD) of respective antimicrobials between the pre-pandemic and pandemic periods at the country level. Horizontal bars represent the lower and upper values of a 95% confidence interval. Black square symbols represent the odds ratio, and red square symbols represent tests with significant differences in the prescription of respective antimicrobials between pre-pandemic and pandemic periods. Antimicrobials with <15 prescriptions overall are excluded from the analysis. Statistical analysis was performed using linear regression. All models adjusted for age (continuous), sex (male/female/other), admitting ward (ICU/HDU/DCC/COVID-specialised [including regular wards for the pre-pandemic period]), comorbidities (binary, yes or no), patient outcome (died/discharged alive) and diagnosis type (sepsis only/pneumonia only/ARDs only/sepsis and pneumonia/sepsis and ARDs/pneumonia and ARDs/sepsis, pneumonia and ARDS).

**Figure S8.** Plot represents the comparison of DOT of respective antimicrobials between COVID-19 positive and COVID-19 negative cases at the country level. Horizontal bars represent the lower and upper values of a 95% CI. Black square symbols represent the odds ratio, and red square symbols represent the significant differences in the prescription of respective antimicrobials between COVID-19 positive and COVID-19 negative cases. The antimicrobials with the <15 prescriptions overall are excluded from the analysis. Statistical analysis was performed using linear regression. All models adjusted for age (continuous), sex (male/female/other), admitting ward (ICU/HDU/DCC/COVID-specialised [including regular wards for the pre-pandemic period]), comorbidities (binary, yes or no), patient outcome (died/discharged alive) and diagnosis type (sepsis only/pneumonia only/ARDS only/sepsis and pneumonia/sepsis and ARDS/pneumonia and ARDS/sepsis, pneumonia and ARDS).

**Figure S9.** Plot represents the comparison of PDD of respective antimicrobials between COVID-19 positive and COVID-19 negative cases at the country level. Horizontal bars represent the lower and upper values of a 95% CI. Black square symbols represent the odds ratio, and red square symbols represent the significant differences in the prescription of respective antimicrobials between COVID-19 positive and COVID-19 negative cases. The antimicrobials with the <15 prescriptions overall are excluded from the analysis. Statistical analysis was performed using linear regression. All models adjusted for age (continuous), sex (male/female/other), admitting ward (ICU/HDU/DCC/COVID-specialised [including regular wards for the pre-pandemic period]), comorbidities (binary, yes or no), patient outcome (died/discharged alive) and diagnosis type (sepsis only/pneumonia only/ARDS only/sepsis and pneumonia/sepsis and ARDS/pneumonia and ARDS/sepsis, pneumonia and ARDS). The difference in sulfamethoxazol/trimethoprim for Switzerland was not shown in the figure as the lower value of CI was zero.

**Figure S10.** Time series analysis of PDD in grams per 100 patients for ceftriaxone prescription in Bangladesh following the emergence of Delta variant. The autocorrelation parameter rho used was based on the autocorrelation of the residuals.

**Figure S11.** Time series analysis of PDD in grams per 100 patients for meropenem prescription in Bangladesh following the emergence of Delta variant. The autocorrelation parameter rho used was based on the autocorrelation of the residuals.

**Figure S12.** Time series analysis of PDD in grams per 100 patients for moxifloxacin prescription in Bangladesh following the emergence of Delta variant. The autocorrelation parameter rho used was based on the autocorrelation of the residuals.

**Figure S13.** Time series analysis of PDD in grams per 100 patients for azithromycin prescription in Bangladesh following the release of WHO guidelines v1. The autocorrelation parameter rho used was based on the autocorrelation of the residuals.

**Figure S14.** Time series analysis of PDD in grams per 100 patients for azithromycin prescription in Brazil following the release of WHO guidelines v1. The autocorrelation parameter rho used was based on the autocorrelation of the residuals.

**Figure S15.** Time series analysis of PDD in grams per 100 patients for azithromycin prescription in India following the release of WHO guidelines v1. The autocorrelation parameter rho used was based on the autocorrelation of the residuals.

**Figure S16.** Time series analysis of PDD in grams per 100 patients for azithromycin prescription in South Korea following the release of WHO guidelines v1. The autocorrelation parameter rho used was based on the autocorrelation of the residuals.

**Figure S17.** Time series analysis of PDD in grams per 100 patients for azithromycin prescription in Brazil following the release of WHO guidelines v2. The autocorrelation parameter rho used was based on the autocorrelation of the residuals.

**Figure S18.** Time series analysis of PDD in grams per 100 patients for sulfamethoxazole/trimethoprim prescription in India following emergence of Delta variant. The autocorrelation parameter rho used was based on the autocorrelation of the residuals.

**Text S1.** **Eligibility criteria of the study participants.**

Patients admitted in ICU, HDU, DCC or specialised COVID isolation wards, with optional transit through emergency department presenting with the following were included in this study.

1. Pneumonia and/or
2. ARDS and/or
3. Sepsis related to any infectious syndrome beyond the respiratory tract (e.g. meningitis, urosepsis, peritonitis, endocarditis, cellulitis etc.)

COVID-19 positivity was not a prerequisite for inclusion, and children aged 18 years or younger were excluded from this study.

**Patients with pneumonia were included based on the following criteria^1^:**

- History of fever followed by respiratory rate of over 20 per minute or pulse rate greater than 100 beats per minute
- Crackles on auscultation, and/or
- Radiological evidence of pneumonia

**Patients with ARDS were included based on the following criteria^2,3^:**

- Bilateral opacities, documented by either chest radiograph or chest CT or ultrasonography, and/or
- The ratio of partial pressure of oxygen (PaO_2_) and fraction of inspired oxygen (FiO_2_) was ≤300 mm Hg or patients with an initial PaO_2_/FiO_2_ of 201–300 mm Hg who were receiving invasive or non-invasive ventilation with a tight-fitting mask and positive end expiratory pressure (PEEP) or continuous positive airway pressure (CPAP) of at least 5 cm H_2_O, or for resource limited setting, if pulse oximetric oxygen saturation (SpO2)/FiO_2_ ratio of ≤315 without the requirement for PEEP, and/or
- Patients treated with high-flow nasal oxygen of 30 L/min

**Patients with sepsis were included based on the following criteria^4^:**

If patients have at least 2 of the following clinical criteria, were included in this study as suspected sepsis cases:

- Altered mentation [Glasgow coma scale (GCS) score of <15)
- Respiratory rate of ≥22/min
- Systolic blood pressure of ≤100 mm Hg

As all our clinical sites reported having their first COVID-19 confirmed case between 20 January 2020 and 02 April 2020, we included patients’ data from 01/10/2019 (or four months prior to index COVID-19 case at the country level, whichever date occurred first) to 30/11/2021, thus the data collection was both retrospective and prospective. Case selection was conducted by the respective clinical sites.

**References**

1. Htun TP, Sun Y, Chua HL, *et al*. Clinical features for diagnosis of pneumonia among adults in primary care setting: A systematic and meta-review. *Sci Rep* 2019; 9: 7600.
2. Thompson BT, Chambers RC, Liu KD. Acute Respiratory Distress Syndrome. *N Engl J Med* 2017; 377: 562-572.
3. Matthay MA, Thompson BT, Ware LB. The Berlin definition of acute respiratory distress syndrome: should patients receiving high-flow nasal oxygen be included? *Lancet Respir Med* 2021; 9: 933-936.
4. Singer M, Deutschman CS, Seymour CW, *et al*. The Third International Consensus Definitions for Sepsis and Septic Shock (Sepsis-3). *JAMA* 2016; 315: 801-10.
